# Do water's electrons care about electrolytes?†
†Electronic supplementary information (ESI) available. See DOI: 10.1039/c8sc03381a


**DOI:** 10.1039/c8sc03381a

**Published:** 2018-11-01

**Authors:** Marvin N. Pohl, Eva Muchová, Robert Seidel, Hebatallah Ali, Štěpán Sršeň, Iain Wilkinson, Bernd Winter, Petr Slavíček

**Affiliations:** a Fritz-Haber-Institut der Max-Planck-Gesellschaft , Faradayweg 4-6 , D-14195 Berlin , Germany . Email: winter@fhi-berlin.mpg.de; b Fachbereich Physik , Freie Universität Berlin , Arnimallee 14 , D-14195 Berlin , Germany; c Department of Physical Chemistry , University of Chemistry and Technology , Technická 5 , 16628 Prague , Czech Republic . Email: petr.slavicek@vscht.cz; d Helmholtz-Zentrum Berlin für Materialien und Energie , Hahn-Meitner-Platz 1 , D-14109 Berlin , Germany . Email: iain.wilkinson@helmholtz-berlin.de; e Humboldt-Universität zu Berlin , Department of Chemistry , Brook-Taylor-Str. 2 , D-12489 Berlin , Germany

## Abstract

Ions have a profound effect on the geometrical structure of liquid water and an aqueous environment is known to change the electronic structure of ions. Here we combine photoelectron spectroscopy measurements from liquid microjets with molecular dynamical and quantum chemical calculations to address the reverse question, to what extent do ions affect the electronic structure of liquid water? We study aqueous solutions of sodium iodide (NaI) over a wide concentration range, from nearly pure water to 8 M solutions, recording spectra in the 5 to 60 eV binding energy range to include all water valence and the solute Na^+^ 2p, I^–^ 4d, and I^–^ 5p orbital ionization peaks. We observe that the electron binding energies of the solute ions change only slightly as a function of electrolyte concentration, less than 150 ± 60 meV over an ∼8 M range. Furthermore, the photoelectron spectrum of liquid water is surprisingly mildly affected as we transform the sample from a dilute aqueous salt solution to a viscous, crystalline-like phase. The most noticeable spectral changes are a negative binding energy shift of the water 1b_2_ ionizing transition (up to –370 ± 60 meV) and a narrowing of the flat-top shape water 3a_1_ ionization feature (up to 450 ± 90 meV). A novel computationally efficient technique is introduced to calculate liquid-state photoemission spectra using small clusters from molecular dynamics (MD) simulations embedded in dielectric continuum. This theoretical treatment captured the characteristic positions and structures of the aqueous photoemission peaks, reproducing the experimentally observed narrowing of the water 3a_1_ feature and weak sensitivity of the water binding energies to electrolyte concentration. The calculations allowed us to attribute the small binding energy shifts to ion-induced disruptions of intermolecular electronic interactions. Furthermore, they demonstrate the importance of considering concentration-dependent screening lengths for a correct description of the electronic structure of solvated systems. Accounting for electronic screening, the calculations highlight the minimal effect of electrolyte concentration on the 1b_1_ binding energy reference, in accord with the experiments. This leads us to a key finding that the isolated, lowest-binding-energy, 1b_1_, photoemission feature of liquid water is a robust energetic reference for aqueous liquid microjet photoemission studies.

## Abstract 2

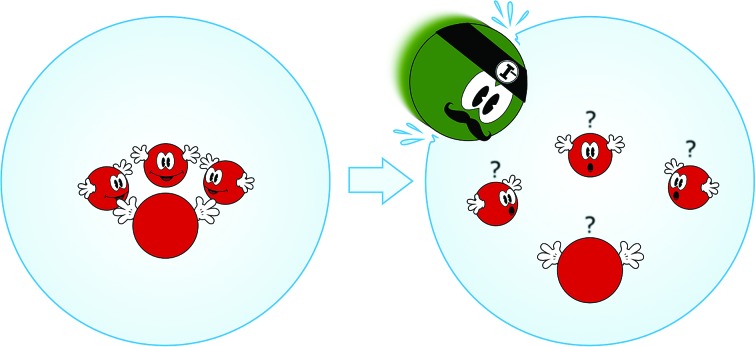
Ions have a profound effect on the geometrical structure of liquid water and an aqueous environment is known to change the electronic structure of ions.

## 1. Introduction

More than a hundred years ago, Arrhenius, Ostwald, and van't Hoff revolutionized chemistry by establishing the ionic theory of electrolytes.^1^ Yet, the molecular understanding of ion–solvent interactions still exhibits surprising gaps.^2^ Even the geometric structure of liquid water itself remains a subject of ongoing controversy, with conflicting views emerging from different experimental and theoretical approaches.^3^–^11^ Still less is known about electrolyte solutions.^12^–^14^

In the present work, we focus on the effects of dissolved atomic ions, particularly Na^+^ and I^–^, at high concentration on the electronic structure of liquid water. The electrolytes are expected to induce significant electrostatic effects and disruptions of hydrogen bonding networks, especially for highly concentrated solutions.^11^,^15^–^17^ The iodide anion has been found to have an exceptional influence on the extended hydrogen bond (HB) network due to its large polarizability, allowing charge to be delocalized to water molecules.^18^ Further understanding of the associated effects on the electronic structure of water are important from a fundamental perspective as well as for applications. For instance, from a practical standpoint, highly concentrated electrolyte solutions extend the water electrochemical window from 1.5 eV to 3.0 eV, prompting recent suggestions to apply such solutions in a variety of safe and environmentally friendly devices, such as aqueous-solution based batteries.^19^–^21^ Also, the (anomalous) increase in screening length that follows the classical decrease of the Debye screening length in dilute electrolytes is of substantial relevance to chemistry, biology, and energy storage.^22^–^24^ Furthermore, as aqueous phase photoelectron (PE) spectroscopy measurements are often performed at relatively high solute concentrations and energy-referenced to the binding energies of liquid water, such screening effects are of great importance to the interpretation of liquid-phase PE spectroscopy measurements, as will be shown in the following text.

The ground-state electronic wave function of isolated water is dominated by a (1a_1_)^2^(2a_1_)^2^(1b_2_)^2^(3a_1_)^2^(1b_1_)^2^ electronic configuration (adopting a *C*_2v_ representation). Within this description, the highest-energy electrons are associated with the 1b_1_ orbital, which is essentially an oxygen p orbital with a nodal plane coinciding with the molecular plane. The lower-lying non-bonding 3a_1_ orbital is again of the oxygen p-orbital type. The 1b_2_ electrons dominantly contribute to the O–H bonds while the inner-valence 2a_1_ orbital has prevailing oxygen 2s orbital parentage. The electrons of isolated, gas-phase water molecules are relatively tightly bound, with the first binding energy (BE) of the neutral molecule occurring at 12.6 eV (X[combining tilde]^2^A_1_, (1b_1_)^–1^), and higher vertical valence BEs occurring at 14.8 eV (Ã^2^A_1_, (3a_1_)^–1^), 18.6 eV (B[combining tilde]^2^B_2_, (1b_2_)^–1^) and 32.6 eV (C[combining tilde]^2^A_1_ (2a_1_)^–1^).^25^–^29^ The electron BEs are shifted by approximately 1.2–1.4 eV to lower values in liquid water, with water-cluster BEs bridging these energetic shifts.^30^–^32^ The distribution of electron BEs is broader in liquid water than for isolated gas-phase molecules due to the continuum of hydration configurations that can be adopted in liquid water at ambient temperatures. However, we cannot interpret the liquid water photoemission spectrum as a shifted and broadened spectrum of the monomer – the intermolecular interactions do measurably alter the electronic structure of the water molecule. These interactions may be conceptually described by considering individual water molecules as “superatomic orbitals”, interacting *via* the bonding and antibonding interactions of their frontier molecular orbitals (1b_1_, 3a_1_, 1b_2_, 2a_1_ – the ‘superatomic orbitals’). This effect is highlighted by the splitting of the 3a_1_ peak of the monomer to form a doublet peak structure in liquid water that cannot be fit with a single Gaussian peak (for such valence features, a Gaussian line shape is commonly adopted as the spectral intensity profiles primarily reflect environmental inhomogeneous and instrumental resolution limit broadening terms^33^–^35^). The observed unusual spectral shape arises from intermolecular orbital interactions, predominantly 3a_1_–3a_1_ interactions between neighboring molecules.^34^–^36^ This peak splitting is known to be less pronounced than for crystalline ice, with the differences in the ice and liquid water spectra interpreted as a consequence of the varying local geometries in the disordered structure of liquid water.^34^

Analogous to intermolecular bonding interactions in liquid water, electrolyte species may have a profound effect on the electronic structure of liquid water. Indeed, at high concentrations, the effect of ions may be stronger than the electrostatic effects of the water molecular dipoles. Moreover, such ionic effects might be specific as different ions have a propensity to adopt different proximities to individual water molecules.^11^ It is noted that the effects of ions on nearby water molecules are considerably greater for gaseous species when compared to liquid water, the latter exhibiting a surprising screening ability that has been demonstrated for various organic and inorganic solutes.^37^ For example, BEs obtained from PE spectroscopy measurements of solvated nucleic acid bases, nucleosides, and nucleotides are almost identical, in contrast to the BEs of the gas phase or micro-hydrated molecules.^38^–^41^ Na^+^ counterions have been shown to have a minor effect on BEs of multiply charged anions, in contrast to the strong effect of protonation of these anionic species.^42^ In addition to the dielectric screening effect of the solvent, the electrostatic attenuation brought about by the ions (*i.e.*, Debye–Hückel screening^43^) must be also explicitly considered. Such screening abilities will depend on the electrolyte concentration; an effect that is poorly explored for extremely high concentrations. Considering all the above-mentioned aspects, it is by no means clear to what extent water's electronic structure can be tuned by electrolyte addition.

Experimentally, the electronic structure of liquid water can be accessed by various means,^13^,^44^–^49^ with several types of X-ray emission spectroscopies and PE spectroscopy being the most powerful techniques.^9^,^44^,^50^–^53^ However, the energy-dispersive PE spectroscopy requirement of a vacuum environment is not readily met with highly volatile samples such as aqueous solutions. Hence, early PE spectroscopy measurements were only possible from highly concentrated aqueous salt solutions, where the electrolyte induced a significant reduction of the water vapor pressure.^54^–^56^ Later, using total electron yield measurements which did not require a vacuum environment or energy-dispersive detection, the (lowest) ionization onsets of several aqueous solutions were determined.^57^–^59^ Because the electron mean free path in water and aqueous solutions is on the order of just a few molecular layers (it has not been determined quantitatively yet^60^), the application of energy-dispersive PE spectroscopy to an aqueous solution required the development of concepts that increase the electron travel length through the water vapor. The first development of this kind was the liquid microjet technique,^51^,^61^ where one exploits the fact that the vapor pressure of the solution rapidly decreases with distance from a liquid jet. When the sample jet has a micrometer-scale diameter, the electrons emitted from the liquid phase can reach a differentially pumped electron detection chamber unperturbed at an increased transfer length of ∼1 mm under typical experimental conditions. Another way to increase the electron transfer length is *via* so-called ambient-pressure PE spectroscopy, where the lower gas-phase pressure results from efficient differential pumping in the vicinity of the irradiated sample.^62^–^64^ With these experimental developments, valence- and core-electron BEs, as well as energies of second-order electrons, have been measured for more than a decade.^33^,^65^ Thus far, absolute energy scale probes of the valence electronic structure of aqueous samples have almost exclusively relied on the liquid microjet technique. Associated valence PE spectroscopy data has recently been reviewed along with recent advances in related theoretical modeling approaches.^60^ However, despite the rapid development of the liquid-jet valence PE spectroscopy technique over the last decade and its application to a wide range of solutions, there is a crucial but almost neglected detail regarding the accuracy of the reported valence electron BEs from aqueous solutions.

In all studies, an assumption was made that the BE of a solute species can be obtained with reference to the peak of the lowest BE feature (1b_1_, 11.16 ± 0.04 eV BE (ref. 33); a value of 11.31 eV has been reported in a later study^66^ but this has no effect on the present work, as further detailed below) of the liquid water PE spectrum. The reason to continue to adopt this assumption is not ignorance, but rather a reflection of several experimental peculiarities of liquid-jet PE spectroscopy. Since the first liquid jet PE spectroscopy measurements^51^,^61^ it was known that a neat liquid water jet will be almost inevitably charged;^67^ some of the underlying contributions to such charging have been discussed.^66^,^68^,^69^ Distinction and quantification of the different charging contributions and their effects on PE spectra is currently beyond our experimental capabilities and awaits dedicated liquid-jet designs. We note that small amounts of electrolyte are generally added to aqueous samples to mitigate such charging effects, and any uncompensated charge would lead to an energetic shift of the whole PE spectrum; the residual effect being equivalent to applying a small voltage to a (conductive) crystalline sample. A notable remaining point is that in the majority of liquid-jet PE spectroscopy instruments, the electric field that exists between a charged liquid jet and the grounded electron detector is ill-defined. A well-known consequence of this effect is that the peak position of the gas-phase water 1b_1_, or any other gas-phase ionizing transition, depends on the given experimental setup, and hence is generally an inappropriate reference for assigning liquid-phase binding energies. This is another issue that was addressed in the early liquid jet PE studies.

In the present work we analyze valence PE spectra of aqueous NaI solutions (ranging from very low to very high concentrations), and we identify and quantify specific (small) differential changes of the electronic structure of liquid water, as opposed to constant (charge-induced) energy shifts of the entire spectrum; see ref. 67. We contrast this goal with a more common objective to interrogate how water affects the electronic structure of solute species, which is typically discussed with reference to gas-phase BEs of the respective solute molecules.^33^ Furthermore, we critically assess the common procedure of energetically referencing aqueous-phase PE spectra by aligning the water 1b_1_ peak to the 11.16 eV BE value for neat liquid water. The observed small but experimentally measurable changes of the water electronic structure are interpreted with *ab initio* and molecular dynamics (MD) simulations, and we discuss the implications for dielectric screening at large electrolyte concentrations. We emphasize that the present study is not designed to verify the accuracy of the value of the water 1b_1_ BE (11.16 ± 0.04 eV).^70^ The actual value of this reference energy is rather irrelevant here as we explore to what extent the energies of other water orbitals as well as the solute orbitals may change with respect to water's lowest ionization energy.

## 2. Experimental approach: photoemission spectroscopy

PE spectra from 0.05 M, 0.5 M, 1.0 M, 2.5 M, 3.0 M, 4.0 M, 5.0 M, 6.0 M, 7.0 M and 8.0 M NaI aqueous solutions were measured from a ∼24 μm diameter vacuum liquid–water jet. Solutions were prepared by dissolving NaI of ≥99% purity (Sigma-Aldrich, #793558) in highly demineralized water (conductivity ∼0.2 μS cm^–1^). The jet velocity was approximately 80 m s^–1^, and the jet temperature was 8 °C. The main reason for using an iodide salt here is that unlike for all other halide ions in water, the lowest electron detachment peak (I^–^ 5p) is well separated from water's lowest ionization energy peak (1b_1_).^60^ Furthermore, in X-ray absorption spectroscopy measurements, of all of the halide anions, iodide has been observed to have the largest effect on the electronic structure of liquid water.^18^ Ionization photon energies of 180 eV/198 eV were applied in relatively surface-sensitive experiments; in this case the inelastic mean free path (IMFP) of the photoelectrons (approximately 169 eV/187 eV respective kinetic energies for water 1b_1_ ionization) is approximately 1 nm, as defined at the 1/e level.^60^,^71^ Two different photon energies were used in several experimental runs, and even at different beamlines, over the course of one year. In order to probe deeper into the solutions and to ensure any observed BE shifts were not due to interfacial effects we used 650 eV photons, corresponding to an approximately 5 nm IMFP.^60^ With this procedure we were able to observe changes in the water valence band BEs when increasing the NaI concentration. The measurements at 198 eV and 650 eV photon energies were conducted at the U49/2 PGM1 undulator beamline of BESSY II at the Helmholtz-Zentrum Berlin. Spectra at 180 eV incident photon energy were measured during the first measurement period at the U41 PGM undulator beamline of BESSY II. In all experiments electrons were detected with a hemispherical electron-energy analyzer at normal angle with respect to the polarization direction of the linearly polarized incident light. A small X-ray focal size, 80 × 22 μm^2^, at the U49 beamline (23 × 12 μm^2^ at the U41), ensured that the gas-phase signal amounted to less than 10% of the total (photo)electron signal during all measurements. The liquid jet was placed at a ∼500 μm distance from the analyzer entrance orifice (500 μm diameter).

The energy resolution of both beamlines was better than 340 meV for 650 eV photon energies, and better than 60 meV for 198 eV energies. For the 180 eV photon energy, the resolution of the U41 beamline was better than 20 meV. The resolution of the electron analyzer was constant with kinetic energy (about 20 meV, at 20 eV pass energy). However, from fitting analyses of the 3a_1_, 1b_2_, and 1b_1_ PE peaks in multiple data sets, we conclude that water peak positions and widths can generally be determined with ±40–60 meV uncertainties in both our ∼200 eV and 650 eV photon energy data sets. Similar uncertainties are obtained for the more intense and spectrally separated solute peaks (Na^+^ 2p and I^–^ 4d). In situations where solute peaks overlap with other features (I^–^ 5p and 5s peaks) and solute concentrations are relatively low, our BE determination uncertainties are dominated by fitting errors in the range of ± 0.04–0.40 eV. This will be shown below in Table 2 when we present the experimental data.

## 3. Simulation protocol: IEDC with Mulliken projection

We studied the effects of ions on the liquid water PE spectra with small cluster models (water heptamers) embedded in a dielectric continuum. To account for the structural variations encountered in solutions, we extracted cluster geometric configurations from molecular dynamics simulations for neat water and 3 M and 8 M NaI solutions. The clusters were extracted as follows: we randomly selected one water molecule and found the closest six water molecules and the closest sodium and iodide ions. For neat water clusters, we simply extracted a central water molecule and six closest neighbors. In total we selected 500 structures for each concentration. We also performed calculations of idealized water pentamers in which the central water molecule is perfectly tetrahedrally solvated (similarly to a previous study^72^), results are provided in the ESI section.†

The MD simulations were performed using the GROMACS 4.6.7 package^73^ with a non-polarizable force field where water was represented by the SPC/E model.^74^ The parameters for the ions^73^ are summarized in Table 1. The simulation box contained (i) 2240 water molecules in a 3.29177 × 3.29177 × 6.19806 nm^3^ box for the neat water simulations, (ii) 2496 water molecules, 155 iodide ions, and 155 sodium ions in a 3.47095 × 3.47095 × 7.14514 nm^3^ box for the 3 M simulations, and (iii) 1540 water molecules, 350 iodide ions, and 350 sodium ions in 3.36427 × 3.36427 × 6.43305 nm^3^ box for the 8 M simulations. The total length of the simulation was 10 ns, the time step for the propagation was set to 1 fs, and 3D periodic boundary conditions were employed. All simulations were performed under a constant pressure of 1 bar which was controlled by the Parrinello–Rahman barostat^75^ with a coupling constant of 1 ps and a constant temperature of 300 K which was controlled by the Nosé–Hoover thermostat^76^,^77^ with a coupling constant of 1 ps. Constraints were applied to all bonds *via* the Lincs algorithm of fourth order.^78^ The van der Waals interactions were truncated at 1.2 nm; the long-range electrostatic interactions were calculated by the particle mesh Ewald method.^79^ The local structure of the solutions is described *via* the tetrahedral order parameter, *q*.^80^ The parameter focuses on the four closest neighbors of the water oxygen atoms and is sensitive only to angular order. The average value of *q* varies between 0 for an ideal gas to 1 for an ideal tetrahedral arrangement. The parameter describing the variance of distances between a central water molecule and its closest neighbors is the translational tetrahedral order parameter, *S*_k_, defined in ref. 81. *S*_k_ is 0 for an ideal tetrahedron; if the configuration deviates from ideal tetrahedrality, *S*_k_ increases and reaches a maximum value of 1 for an ideal gas.

**Table 1:** Lennard-Jones parameters used in the classical MD simulations for I^–^ and Na^+^ ions,^82^ water was represented by the SPC/E model^72^

	*R*_min_/2 [Å]	*ε* [kcal mol^–1^]
I^–^	2.919	0.0427845
Na^+^	1.212	0.3526418

The BEs for all clusters were calculated with a recently introduced ionization-as-an-excitation-into-a-distant-center (IEDC) approach.^83^ Briefly, the method is based on modelling the ionization from a selected orbital space as an excitation into a continuum using time-dependent density functional (TDDFT) theory. As DFT is in principle an exact many-body theory, we can obtain correlated orbital energies.^84^ In all calculations the long-range corrected Perdew–Burke–Ernzerhof functional (LC-*ω*PBE)^85^ was employed with the range-separation parameter, *ω*, set to 0.45 a_0_^–1^, which was optimized for the water clusters.^31^

We specifically focus on the electronic structure of the fully solvated central water unit. For this purpose we employ the Mulliken-type population analysis^86^ to evaluate the fraction of the selected moiety, *f*_Fr_, contributing to each of the orbitals:1
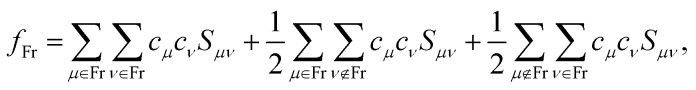
where *S*_*μν*_ is an element of the overlap matrix, *c*_*μ*_ and *c*_*ν*_ are the expansion coefficients, and Fr denotes the fragment of interest (the central water molecule). The spectrum is then modelled *via* a reflection principle approach,^87^,^88^ *i.e.*, the distribution of binding energies along the classical MD trajectory:2
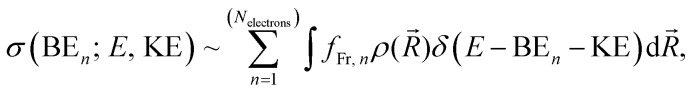
where *ρ*(*R[combining right harpoon above]*) is the nuclear density evaluated with the classical MD simulations, BE_*n*_ is the binding energy of the ejected electron, KE is the kinetic energy of an ejected electron, *E* is the incident photon energy, and *f*_Fr,*n*_ is the contribution from the *n*-th electron of the fragment of interest. In the modelled photoemission spectra we included solvent spectral broadening *via* the reflection principle with an additional broadening scheme (RP-AB),^89^*i.e.*, each point is broadened with a Gaussian function with a variance reflecting the reorganization energy calculated by means of dielectric continuum methods.

As the models used are of a rather limited size, we account for the remaining water molecules *via* the dielectric continuum model represented by a conductor-like polarizable continuum (C-PCM).^90^–^92^ We use the non-equilibrium model of solvation (NEPCM) for the ionized states, *i.e.*, only the fast part of the dielectric constant of water (the “optical” dielectric constant is 1.78) follows ionization while the slow part remains unchanged as in the ground state.^93^–^95^ We employed the state specific (SS) scheme as implemented in the Q-Chem 4.1 code.^96^ We note here that inclusion of the solvation effects in the ionization potential theorem is non-trivial. The embedding of the non-equilibrium response of the solvent to the Kohn–Sham orbital energies has been a subject of a debate, so far attempts were made mainly in different directions.^97^,^98^ The IEDC approach is fully consistent in this respect.^83^

The use of a dielectric continuum model parameterized for water should be further discussed. Indeed, the screening ability of electrolyte solutions is different from that of water.^99^–^102^ Since the electrolyte solutions are conductive, the use of the concept of dielectric screening cannot be fully justified. However, a frequency-dependent permittivity constant is still defined. The low frequency limit of the real part yields the static permittivity (dielectric constant, *ε*_r_) and its value for a wide range of salts decreases as the electrolyte concentration is increased; a phenomenon called a dielectric decrement. The phenomenon is attributed to excluded volume effects and to the formation of hydration shells around ions and ion pairs (ion dipoles) which prevents orientation of these water molecules in the external electric field. The dielectric properties of electrolytes are still a subject of independent research, far beyond the scope of the present work. In our work we adopted a pragmatic approach, correcting the permittivity for concentrated solutions according to experimental and model data for NaCl (data for NaI are not available in the investigated concentration range^103^,^104^) that were extrapolated to high concentrations.^105^,^106^ The permittivity for an 8 M solution has to be taken as approximate. The 3 M and 8 M solution permittivity was set to, *ε*_r_ = 46 and *ε*_r_ = 22, respectively, compared to *ε*_r_ = 78.39 for pure water. The optical dielectric constant, *ε*_opt_, was set according to the dependence of the refractive index on salt concentration^107^ to 1.78 for pure water, 1.86 for the 3 M solution, and 2.01 for the 8 M solution.^108^,^109^

The nature and characteristics of hydrogen bonding in clusters in a polarizable continuum were described in terms of a natural bond orbital^110^ (NBO) analysis. In this scheme, the orthogonal natural orbitals are constructed as eigenvalues of the density matrix obtained from the converged SCF calculation. The orbitals are classified in terms of Lewis structures as bonding, lone pairs, core (for occupied orbitals), and Rydberg and antibonding (for unoccupied orbitals). The donor–acceptor character of hydrogen bonds can be described in terms of a charge transfer from a high-energy occupied orbital of donor (lone pair of oxygen for water, lone pair of the iodide anion) to an unoccupied orbital of the acceptor unit (σ*(O–H) orbital for water^111^). The electronic occupancy of the σ*(O–H) orbital can be used as a possible parameter for quantification of the hydrogen bonding.

## 4. Experimental results

**Fig. 1:**
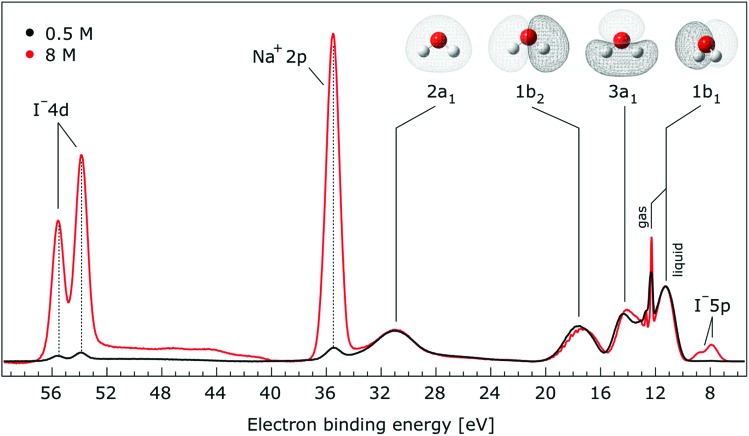
Valence photoelectron spectra of 0.5 M (black) and 8.0 M (red) NaI (aq.) measured at a 198.0 eV photon energy at the U49 beamline. A linear background was subtracted from both spectra in order to account for the contributions of the inelastically scattered electrons. Both spectra appear to be closely energetically aligned at the Na^+^ 2p and I^–^ 4d peaks after shifting the peak of the liquid water 1b_1_ peaks to 11.16 eV, as indicated by dashed lines. Small plateau features in the 41–52 eV BE region for the 8 M solution can be assigned to energy loss processes where Na^+^ 2p photoelectrons excite quasi-optical transitions of water.^112^ Schematics of the valence molecular orbitals of water are reprinted from ref. 139 in the top right of the figure.

In Fig. 1, we present PE spectra from 0.5 M and 8.0 M NaI aqueous solutions measured at a 198 eV incident photon energy, covering the 5–60 eV BE range. This data was recorded at the U49 beamline with a focal spot size of the order of the liquid jet dimensions. At this photon energy, this resulted in the largest gas-phase water spectral contribution of all of the spectra reported here. Both spectra in Fig. 1 have been shifted in energy to match the established 11.16 eV lowest vertical ionization energy (1b_1_) of liquid water;^60^ this procedure by which the 1b_1_ energy is an inherently fixed reference energy, not subjected to any change upon concentration variation is explained in the next paragraph. In addition, we subtracted a linear background signal to minimize contributions from inelastically scattered electrons. The relative intensities of the two spectra are set to yield the best overlap of the 1b_1_ liquid water spectral contributions. Peaks at 13.5 eV, 17.3 eV, and 30.9 eV BE, primarily corresponding to ionization of the liquid water 3a_1_, 1b_2_, and 2a_1_ orbitals, respectively, are in agreement with previous work.^33^ The large peak at 35.0 eV BE arises from ionization of the Na^+^ 2p orbital. Peaks near 8.0 eV and 55.0 eV BEs are due to ionization of the I^–^ 5p_1/2, 3/2_ and I^–^ 4d_3/2, 5/2_ orbitals, respectively.^51^,^60^ Observed relative peak intensities in Fig. 1 reflect solute concentrations weighted by relative partial photoionization cross-sections, the PE angular distributions of the respective water and solute ionization processes, and the electron collection/detection geometry.

We find, from Fig. 1, that when the liquid 1b_1_ peaks are energetically aligned, all other water peaks, as well as the solute peaks, are close to aligned for the two solution concentrations. This good overlap suggests that our choice of a fixed 1b_1_ energy reference is reasonable but at the same time our procedure imposes an inability to quantify any possible energy shifts of the water 1b_1_ orbital. Unfortunately, there is little to improve as any other treatment of the as-measured spectra – affected by liquid charging, true and apparent (scattering-related) BE shifts – would require careful quantification and correction of electrolyte-concentration-dependent sample-analyzer electric field gradients and potentially theoretical corrections for scattering effects, both of which would be subject to relatively large uncertainties. In fact, a strong argument in favor of the spectral alignment at the water 1b_1_ peak is that the best overlap of all peak positions considered in the spectra, including all solute and water peaks, is achieved in this case. That is, the sum of all spectral shifts is smallest with this spectral alignment procedure. We now show that small energetic shifts of the solute peaks are indeed in good agreement with an aligned water 1b_1_ peak. Furthermore, our experimental observations, based on the fixed water 1b_1_ energy, are well corroborated by our computations.

**Fig. 2:**
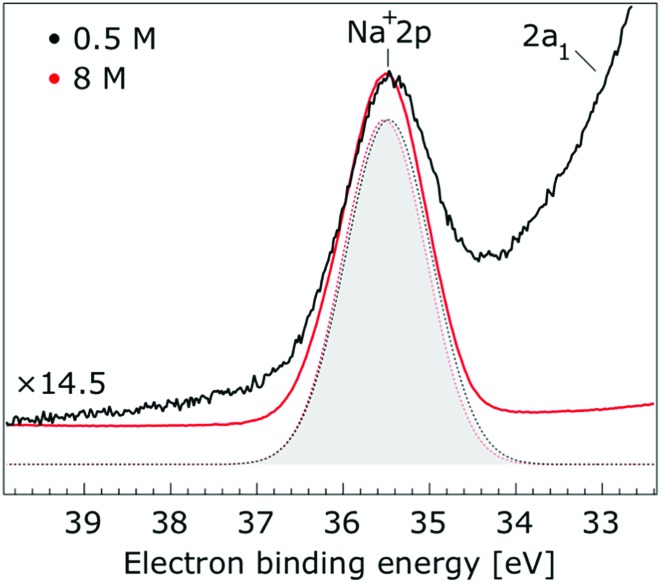
Enlarged view of the Na^+^ 2p and water 2a_1_ photoelectron spectra of 0.5 M (black) and 8.0 M (red) NaI (aq.) solutions measured at a 198 eV photon energy. The intensity of the 0.5 M concentration spectrum was multiplied by 14.5 to yield the same peak height as that observed from the 8.0 M solution. Gaussian fits of the Na^+^ 2p peak are presented (the fits in black and red correspond to the 0.5 M and 8.0 M solutions, respectively). Spectral contributions primarily associated with water 2a_1_ ionization have been subtracted.

**Fig. 3:**
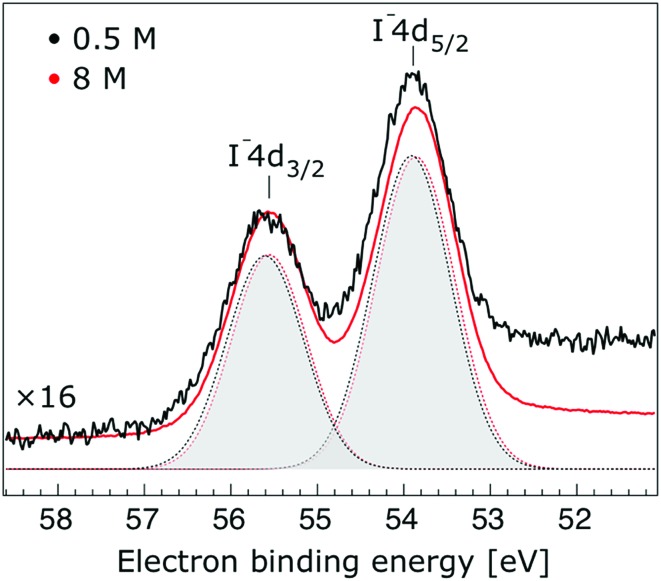
Enlarged view of the I^–^ 4d_5/2_ and I^–^ 4d_3/2_ photoelectron spectra of 0.5 M (black) and 8.0 M (red) NaI (aq.) solutions measured at a 198 eV photon energy. The intensity of the 0.5 M concentration spectrum was multiplied by 16 to yield the same peak heights as those observed from the 8.0 M solution. Gaussian fits of the I^–^ 4d_5/2_ and water I^–^ 4d_3/2_ components are presented (the fits in black and red correspond to the 0.5 M and 8.0 M solutions, respectively). A broad, flat Gaussian background is subtracted from both data sets in order to account for the electron inelastic scattering plateau at the low-KE side of the I^–^ 4d_5/2_ peak.

**Fig. 4:**
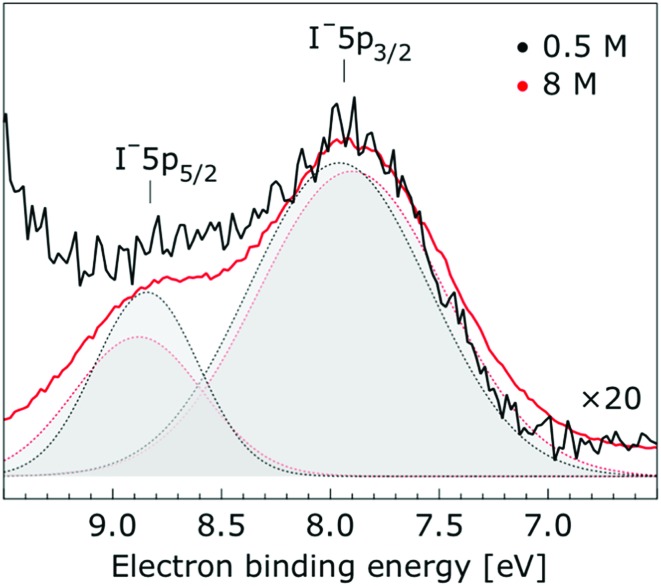
Enlarged view of the I^–^ 5p_3/2_ and I^–^ 5p_1/2_ photoelectron spectra of 0.5 M (black) and 8.0 M (red) NaI (aq.) solutions measured at a 198 eV photon energy. The intensity of the 0.5 M concentration spectrum was multiplied by a factor of 20 to yield the same peak heights as those observed from the 8.0 M solution. Gaussian fits of the I^–^ 5p_3/2_ and water I^–^ 5p_1/2_ components are presented (the fits in black and red correspond to the 0.5 M and 8.0 M solutions, respectively).

Thorough spectral analysis of Fig. 1 (simultaneous fitting of all spectral features) reveals a small energetic shift of the water 1b_2_ peak (with respect to the 1b_1_ peak) and a change of the spectral shape of the water 3a_1_ peak. A detailed analysis of the water 2a_1_ peak will not be presented here; as shown in Fig. 1 this peak position is well-aligned at low and high concentrations. The subtler effects on the solute spectral features are more clearly seen in Fig. 2–4, which present enlarged views of the two Na^+^ 2p (aq.) spectra (Fig. 2), I^–^ 4d (aq.) spectra (Fig. 3), and I^–^ 5p (aq.) spectra (Fig. 4). Fig. 2–4 also show respective Gaussian fits to the data; in the case of Na^+^ 2p (aq.), the 0.5 M spectrum has been subtracted from the 8.0 M spectrum to remove the contributions from the water 2a_1_ electrons. Under the aforementioned conditions, we find that the respective Gaussian peak positions are shifted to slightly lower BEs with increasing concentration (maximum energetic shifts of 110 ± 70 meV with variances in the peak widths < 30 ± 50 meV). The energetic shifts are significantly less than those of the water 1b_2_ feature discussed below. We summarize the obtained experimental BEs of the solute orbitals in Table 2 (Top) where we also present similar data for crystalline NaI;^113^ the valence I^–^ 5p orbitals form the valence band in the crystalline phase and hence, their BEs are not included. We emphasize that all reported BE values correspond to aqueous ground neutral state – aqueous cation/free electron state energy gaps, as referenced to the best of our experimental capabilities to the vacuum level, and that experimental differentiation between initial and final state contributions to any electrolyte-induced BE shifts are not possible.

**Table 2:** (Top) Orbital energies of NaI (aq.) extracted from the vacuum/liquid-interface-sensitive measurements reported in this study (180/198 eV photon energies), and from crystalline NaI.^113^ (Bottom) Orbital energies of NaI (aq.) inferred from the bulk-sensitive measurements reported in this study

	Peak	BE [eV]			
		0.5 M	8.0 M	Δ*E*	NaI crystal
Interface	I^–^ 5p_3/2_	7.83 ± 0.04	7.72 ± 0.05	–0.11 ± 0.07	—
	I^–^ 5p_1/2_	8.70 ± 0.11	8.67 ± 0.05	–0.03 ± 0.12	—
	Na^+^ 2p	35.39 ± 0.04	35.41 ± 0.04	+0.02 ± 0.06	29.29
	I^–^ 4d_5/2_	53.84 ± 0.04	53.74 ± 0.04	–0.10 ± 0.06	47.68
	I^–^ 4d_3/2_	55.52 ± 0.04	55.44 ± 0.06	–0.08 ± 0.07	49.35
Bulk	I^–^ 5p_3/2_	7.82 ± 0.04	7.71 ± 0.04	–0.11 ± 0.06	—
	I^–^ 5p_1/2_	8.71 ± 0.10	8.67 ± 0.04	–0.04 ± 0.11	—
	I^–^ 5s	Not detectable	19.6 ± 0.4	Not detectable	11.21
	Na^+^ 2p	35.44 ± 0.04	35.41 ± 0.04	–0.03 ± 0.06	29.29
	I^–^ 4d_5/2_	53.89 ± 0.04	53.74 ± 0.04	–0.15 ± 0.06	47.68
	I^–^ 4d_3/2_	55.57 ± 0.04	55.44 ± 0.04	–0.13 ± 0.06	49.35

**Fig. 5:**
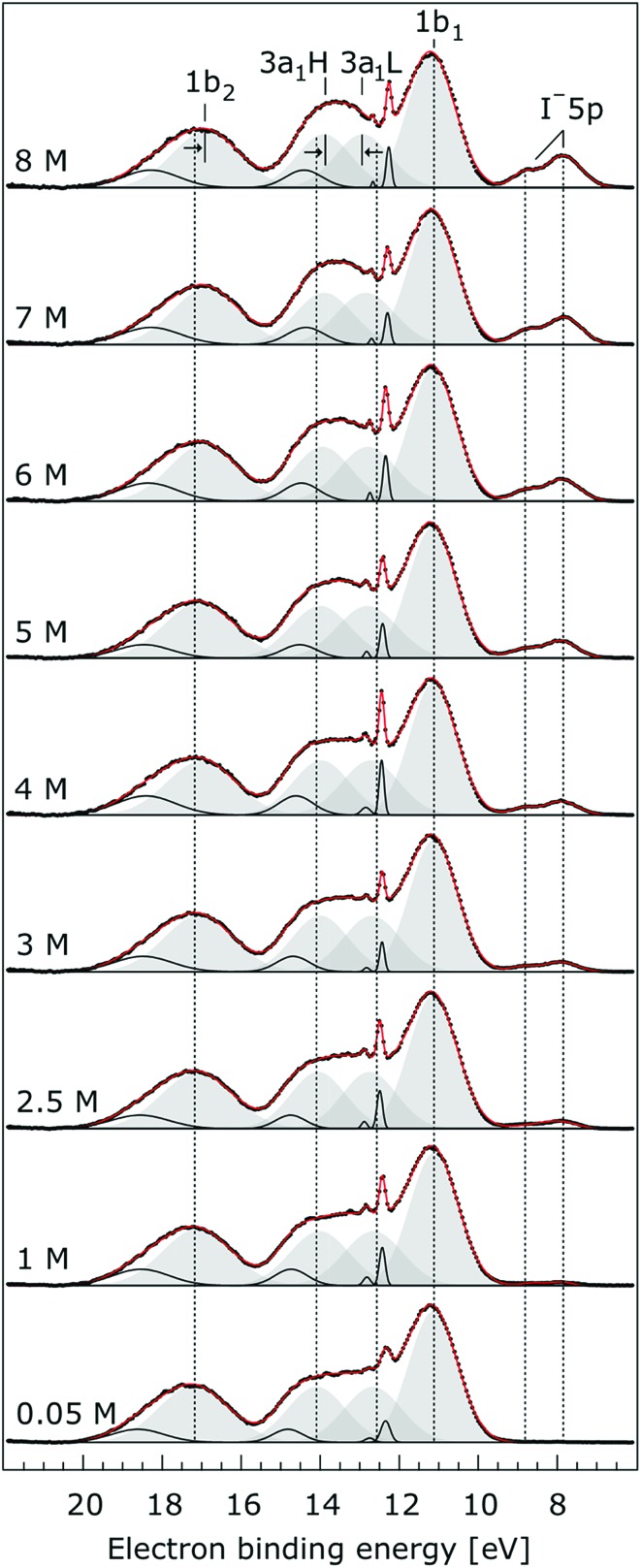
Valence photoelectron spectra from NaI aqueous solutions as a function of concentration, 0.05 M to 8.0 M. The high energy resolution spectra were recorded at the U41 beamline with a photon energy of 180 eV. Intensities are displayed to yield the same liquid water 1b_1_ peak heights for each solution. Peaks in grey are the Gaussian fits representing the photoelectron contributions primarily due to ionization of the four liquid water valence orbitals. Solid black lines are Gaussian fits of gas-phase water signal contributions. For the lowest concentration, the fit parameters previously reported for water^32^ were used. For the higher concentrations, the energetic positions and widths of all peaks were allowed to vary. Both 3a_1_ Gaussian fit component peaks (3a_1_ L and 3a_1_ H, see the main body of the text for details) are constrained so that they exhibit similar widths and heights in each spectrum.

**Fig. 6:**
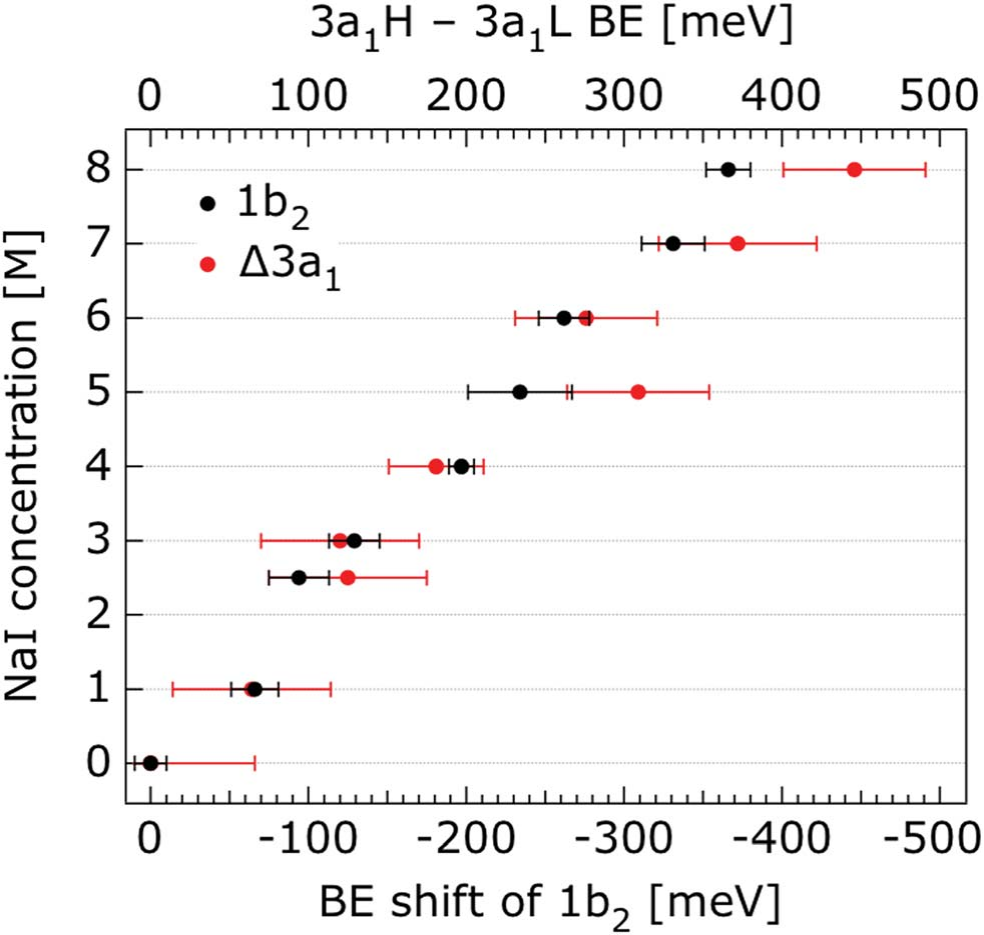
Decrease of the water 1b_2_ binding energy (with respect to that of 1b_1_), and decreasing 3a_1_ splitting in NaI aqueous solutions as a function of concentration, 0.05 M to 8.0 M. All data were extracted from Fig. 5. The error bars represent the uncertainties of the fitting procedure.

In order to quantify the small spectral changes of the water valence band, which displays significant overlap among the spectral peaks, we consider the series of spectra spanning 0.05 M to 8.00 M NaI concentrations shown in Fig. 5. These spectra were recorded with a 180 eV photon energy, a higher energy resolution, and a smaller focal spot size at the U41 beamline (see the Experimental approach section), resulting in lower gas-phase spectral contributions with respect to Fig. 1. The 0.05 M solution is representative of neat water, with the fairly small amount of salt serving to achieve sufficient electric conductivity for high-acquisition-rate PE spectroscopy experiments.^51^ Note that over the large concentration variation, from 0.05 M to 8.00 M, the viscosity of the aqueous solution increases, potentially leading to the liquid jet experiencing small (μm) changes in position which will alter the relative liquid-to-gas signal intensity ratio. With the water 1b_1_ peaks aligned to 11.16 eV, a shift to lower BE up to 370 ± 60 meV is observed for the water 1b_2_ peak across the concentration range shown in Fig. 5. The evolution of the 1b_2_ peak shift, as obtained from Gaussian fits, is displayed in Fig. 6. Indicated error bars were determined from uncertainties of the fitting procedure. A 1b_2_ peak-width analysis, presented in Fig. SI-1 in the ESI,† reveals no noticeable trends, and this quantity is not further considered here.

The other, and more notable, spectral change occurs for the water 3a_1_ peak, in the 12–16 eV BE region. At low concentrations, the flat-top profile typical for neat liquid water, represented by the two aforementioned Gaussians, is observed. (Note that the 3a_1_ flat-top profile is obscured in Fig. 1 due to overlap with gas-phase spectral contributions.) With increasing concentration, the 3a_1_ flat-top feature evolves into a broad maximum. The 3a_1_ feature comprises of two orbital components that are primarily associated with intermolecular bonding and non-bonding interactions between water molecules, and have been formerly referred to as the 3a_1_ L and 3a_1_ H bands, respectively.^35^,^66^ Accordingly, we consider the 3a_1_ peak change with concentration a consequence of a varying energetic spacing between the 3a_1_ L and 3a_1_ H bands. To quantify such an effect, the 3a_1_ peak profiles were fit using a pair of Gaussian components, starting with the 0.05 M NaI solution, and using the well-established water peak widths and energies.^33^ Fixing the 3a_1_ L and 3a_1_ H peak widths to equal values and ensuring equal spectral contributions to the fit, we find that a small shift to higher BEs of the 3a_1_ L peak, and a small shift to lower BEs of the 3a_1_ H peak nicely represents the evolving overall 3a_1_ peak shape. In going from a 0.05 M to 8.00 M concentration, the 3a_1_ peak-splitting reduces by 450 ± 90 meV. The evolution of the 3a_1_ peak-splitting is also shown in Fig. 6A and is of similar magnitude to the concentration-dependent differential peak shift observed for the 1b_2_ PE features.

**Fig. 7:**
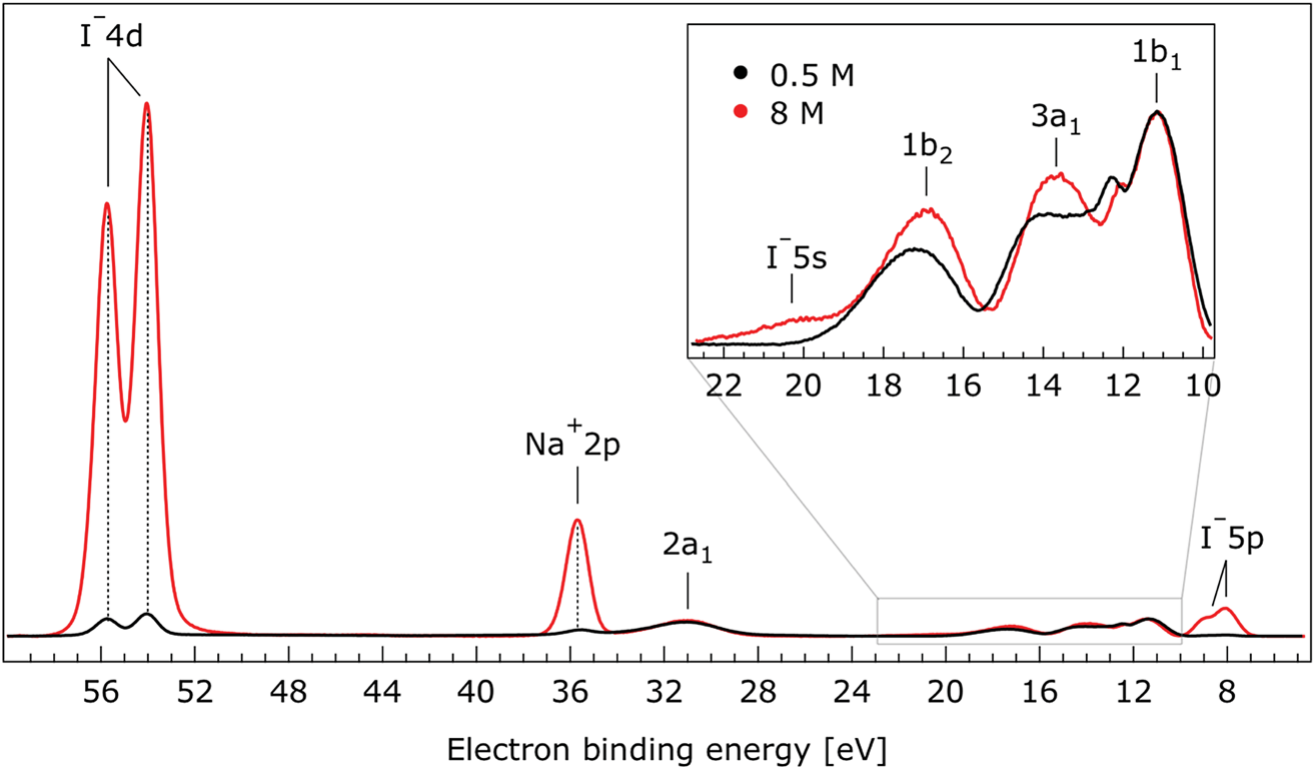
Valence photoelectron spectra of 0.5 M (black) and 8.0 M (red) NaI (aq.) measured at a 650.0 eV photon energy. Inset: enlarged region associated with the outer-valence spectrum.

So far, the experimental results have been presented for the rather low photon energies 180 eV and 198 eV which produce photoelectrons with relatively short IMFPs,^60^ allowing us to primarily probe the liquid–vacuum interface. Our previous liquid-jet PE studies on the dissociation of HNO_3_ at the aqueous solution surface^114^ or on the molecular propensity of amine aqueous solutes with different dominant protonation states to concentrate at an interface^115^ highlighted that uniquely interfacial behavior can be detected when electrons are produced with kinetic energies near 100 eV. This finding is consistent with the shortest electron IMFPs in water,^71^,^116^ which is believed to be approximately 1 nm, for energies in the approximately 50–200 eV range. When increasing the kinetic energy by ∼400–500 eV one rather obtains spectra characteristic of the bulk aqueous solution. In order to ascertain whether the observed spectral changes discussed so far are properties of the interface, we have also measured the valence PE spectra at a 650 eV photon energy, corresponding to an approximate electron IMFP of 5 nm.^117^,^118^ We show the results from the 650 eV photon energy measurements in Fig. 7.

The most important observation from Fig. 7 is that the solute and water BEs do not change significantly with increasing salt concentration in these bulk-sensitive measurements; similar to the interfacially-sensitive results shown in Fig. 1. However, upon close inspection, slight shifts to lower BE of the solute peaks of up to 150 ± 60 meV are observed with increasing concentration in the bulk sensitive data. The associated shifts can be seen more clearly in the enlarged views of the Na^+^ 2p, I^–^ 4d, and I^–^ 5p BE regions shown in Fig. SI-2 to SI-4,† respectively. The positions and concentration-dependent peak shifts of the individual solute peaks are also summarized in Table 2 (bottom). Considering the water PE spectrum features, the 1b_2_ and 3a_1_ peaks are observed to display qualitatively similar behavior in the aqueous bulk as at the vacuum–liquid interface. From Gaussian fits to the 1b_2_ PE features in Fig. 7, a 330 ± 60 meV shift to lower BE is extracted when the NaI concentration is raised from 0.5 M to 8.0 M. Similarly to the analysis of the data shown in Fig. 5, pairs of constrained Gaussians were fit to the 3a_1_ features in the 0.5 M and 8.0 M spectra. As the concentration was increased, the 3a_1_ L and 3a_1_ H fit components were found to shift to higher and lower energies, respectively, resulting in a narrowing of the peak separation by 310 ± 120 meV. Collectively considering the concentration-dependent BE changes extracted from Fig. 7, we conclude that equivalent results are obtained from the vacuum–liquid interface and aqueous-bulk-sensitive measurements. This is an interesting finding, suggesting that differences in the interfacial and bulk-solution structure have no (detectable) effect on the spectral positions of the reported valence PE spectra.

## 5. Theoretical results

**Fig. 8:**
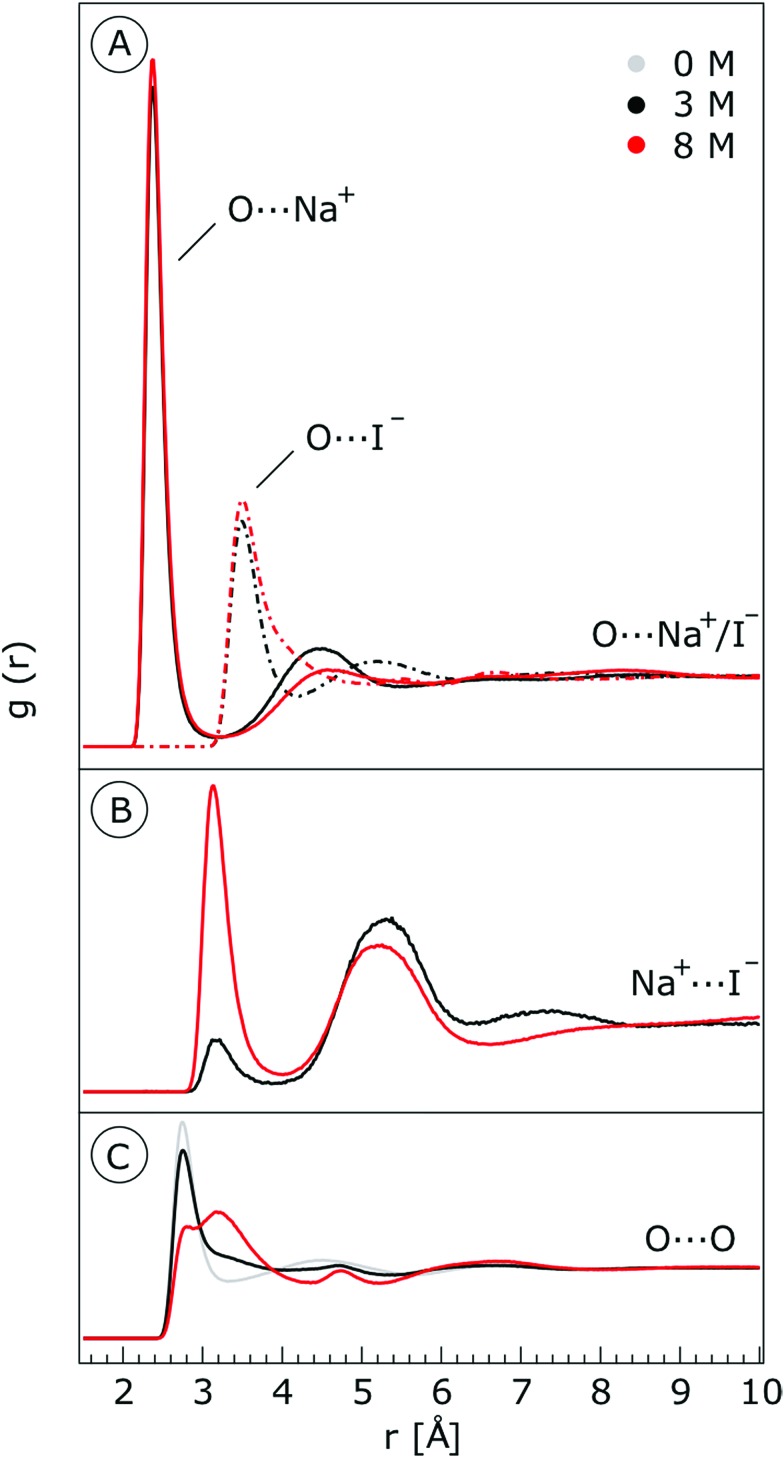
Radial distribution functions for (A) water O–Na^+^ (solid lines) and water O–I^–^ (dashed lines) and (B) Na^+^–I^–^ in the 3 M and 8 M aqueous solutions of NaI. (C) Radial distribution functions for O–O for neat water (labelled 0 M), 3 M and 8 M aqueous solutions of NaI.

We characterize the molecular structure of the highly concentrated solutions with classical molecular dynamics (MD) simulations. We start by considering the radial distribution functions (RDFs) in the aqueous solutions; see Fig. 8. Note that all the presented results correspond to molecules in the bulk, *i.e.*, we do not focus on the vacuum–liquid interface. The first peak of the O–Na^+^ RDF shown in Fig. 8A occurs at 2.37 Å; the first peak for the O–I^–^ RDF occurs at 3.50 Å. Both are in good agreement with previous MD studies.^82^,^119^ The Na^+^–I^–^ RDF shown in Fig. 8B exhibits two peaks and a broad third peak. The first one at ∼3 Å corresponds to the contact ion pair, the second at ∼5 Å corresponds to the solvent-shared ion pair configurations. The third broad peak corresponds to the solvent-shared ion pair, which is typically observed for salt solutions. The precise fraction between contact-ion pairs and solvent-shared ion pairs is a point of ongoing debate, with distinct results obtained for the different force field models used.^82^,^120^,^121^ The total fraction of ion-pair structures, however, undoubtedly increases significantly when passing from 3 M to 8 M solution. We also show the RDF for O–O in Fig. 8C. As can be inferred from the figure, the water structure is slightly altered in the 3 M solution (similar to previous findings, *e.g.*, in ref. 122). However, for the 8 M solution, the RDF for O–O is dramatically different. We have to note here that the MD simulations do not necessarily provide a quantitatively correct evaluation of the liquid molecular structure for the highly concentrated solution, but the observed trends are still relevant.

For a 3 M solution, the coordination numbers of the ions around water are 0.450 for an iodide anion and 0.329 for a sodium cation (see Table 3). The coordination numbers are calculated as 
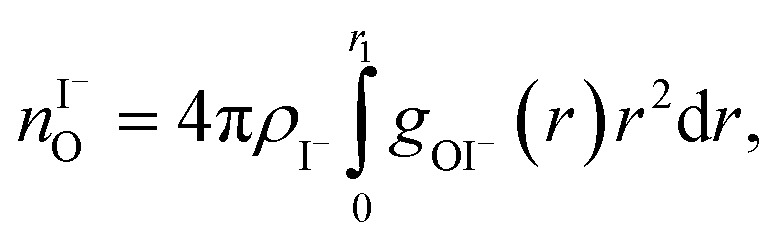
 where *ρ*_I^–^_ is the I^–^ number density, *g*_OI^–^_ is the radial distribution function of O–I^–^ with the first minimum at *r*_1_. Analogous O–Na^+^ calculations were also performed. The tetrahedral order parameter and translational tetrahedral order parameters are collected in Table 3 as well. As can be inferred from the table, both parameters (*q* and *S*_k_) indicate that the 3 M solution structure has a less tetrahedral character compared to bulk water (*q* decreases and *S*_k_ increases). The structures of the 500 (H_2_O)_7_Na^+^I^–^ clusters extracted from the simulations reflect bulk properties; the sodium cation is within the first coordination shell (distance smaller than 3.2 Å from the central water molecule) for 33% of structures, yet only 15% of the structures have sodium coordinated in a tetrahedral position (angle H–O···Na^+^ in the range 109° ± 20°). The iodide anion is within the first coordination shell (at distances less than 4.5 Å from the central water molecule) for 44% of the structures and in 27% of the structures the anion forms a HB with the central water molecule (O–H···I^–^ in the range 180° ± 20°). Less than 5% of the structures have both sodium and iodide ions tetrahedrally coordinated in the first coordination shell. For the 8 M solution, the coordination numbers are 1.00 for Na^+^, and 2.69 for I^–^. Therefore, a randomly selected water molecule has a sodium cation in its first coordination shell at a distance less than 3.2 Å from the central water molecule. The first minimum on the O···I RDF is localized at a distance of 4.5 Å; *i.e.*, the coordination shell is larger than for Na^+^ and more than two iodide anions are typically found within the shell. Both order parameters in Table 3 show that the tetrahedrality of the solution further decreases compared to the 3 M solution, the local structure of the solution is clearly affected by the ions. Here again, the 500 clusters extracted from the MD simulations reflect the bulk structural properties, 80% of (H_2_O)_7_Na^+^I^–^ structures have a sodium cation within 3.2 Å and 100% of the structures have an iodide anion within 4.5 Å. 35% of the structures have a sodium cation tetrahedrally coordinated to the central water molecule, 55% of the structures have the iodide anion hydrogen-bonded to the central water molecule, and 20% of all structures have both iodide and sodium ions coordinated tetrahedrally in the first solvation shell.

**Table 3:** Mean coordination numbers of water in NaI solutions, mean values of order parameters (orientational tetrahedral parameter *q* and translational tetrahedral parameter *S*_k_) and the average number of hydrogen bonds per water molecule. The coordination numbers were calculated as 
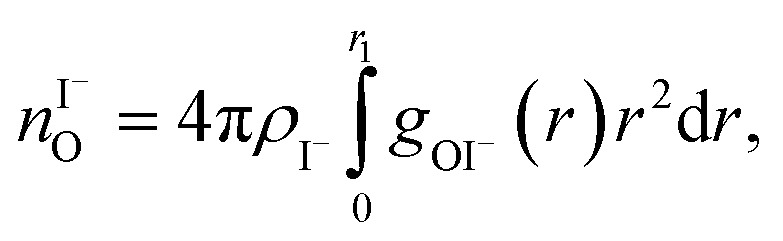
 where *ρ*_I^–^_ is the I^–^ number density, *g*_OI^–^_ is the radial distribution function of O–I^–^ with the first minimum at *r*_1_. The O–Na^+^ mean coordination numbers were analogously calculated. The position of the first minima for I^–^ for 3 M concentration is 4.5 Å and for 8 M it is 5.04 Å, which explains a dramatic increase in coordination number. For Na^+^, the increase in the coordination number corresponds to the increase in concentration; the position of the first minima is 3.2 Å for both 3 M and 8 M

Concentration	Coord. number I^–^	Coord. number Na^+^	*q*	*S*_k_	Number of H-bonds/molecule
0 M	—	—	0.6300(0.0050)	0.00122(0.00003)	3.58
3 M	0.450	0.329	0.5076(0.0051)	0.00205(0.00003)	2.73
8 M	2.691	1.001	0.3266(0.0076)	0.00326(0.00004)	1.16

The average number of HBs formed per water molecule with other water molecules is collected in the last column of Table 3. We follow a geometrical definition of a HB, *i.e.*, the O···H distance is smaller than 2.3 Å and the O_*j*_ –O_*i*_···H_*i*_ angle is smaller than 30°. The higher number of HBs in neat water reflects the fact that ions substitute tetrahedrally coordinated water; the number therefore decreases with increasing the electrolyte concentration.^123^ For the 8 M solution, the calculated average number is only 1.16 per water molecule.

**Fig. 9:**
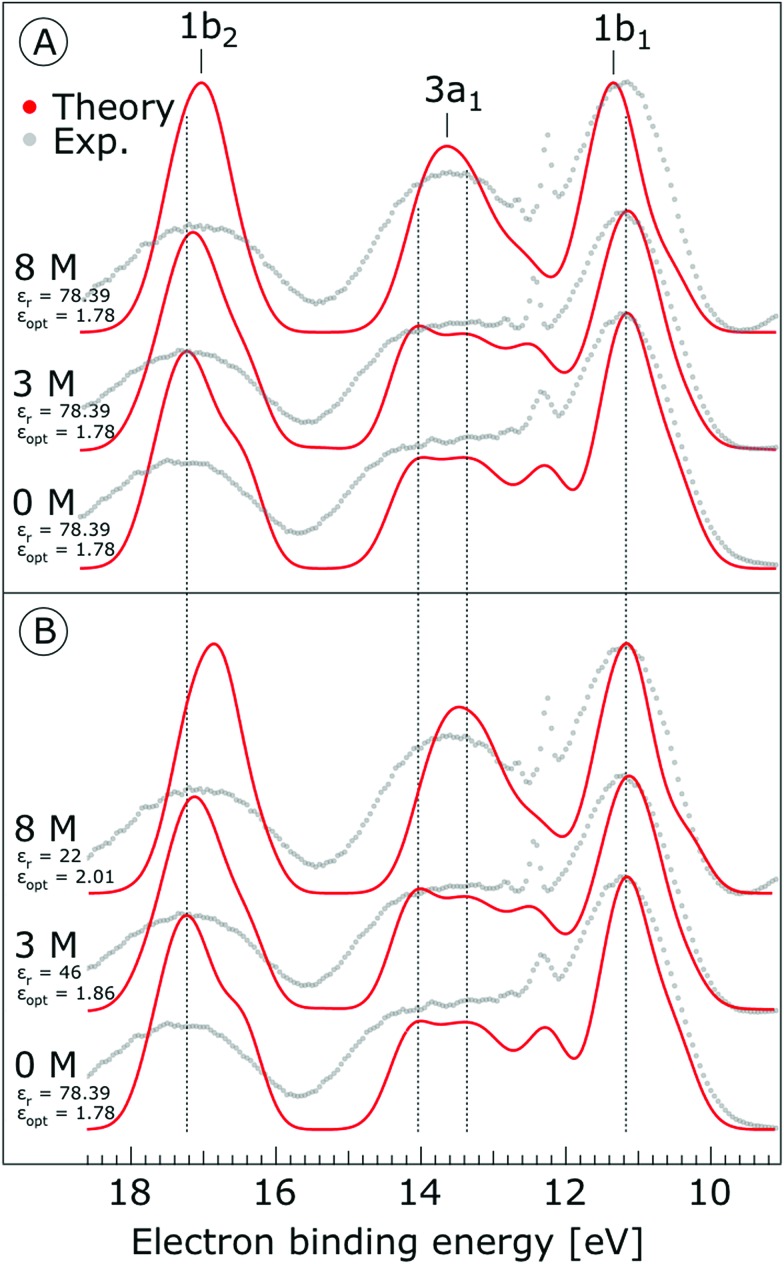
Theoretical photoemission spectra. (A) Simulations for water heptamer clusters containing one Na^+^ and one I^–^ ion embedded in a polarizable continuum having a permittivity, *ε*_r_, of 78.39 (which corresponds to pure water) and *ε*_opt_ = 1.78. (B) Simulations for the same water clusters embedded in a polarizable continuum having a permittivity *ε*_r_ = 46 and *ε*_opt_ = 1.86 for the 3 M NaI, and *ε*_r_ = 22 and *ε*_opt_ = 2.01 for the 8 M NaI solution. Data for water (labelled ‘0 M’) corresponds to water heptamers embedded in a polarizable continuum and having a permittivity of 78.39 in both (A) and (B). Experimental spectra are aligned at the water 1b_1_ peak (11.16 eV BE). All theoretical spectra are shifted to lower energies by 0.3 eV, so that the spectra representing pure water are aligned at the known 1b_1_ BE as well.

The photoemission spectra were calculated for 500 representative (H_2_O)_7_Na^+^I^–^ (or (H_2_O)_7_ for neat water) clusters extracted from the MD simulations. We present the calculated PE spectra for neat water, 3 M and 8 M NaI aqueous solutions in Fig. 9. Panels A and B show the PE spectra obtained for different dielectric continuum permittivities. In order to explore the effect of dielectric screening, the spectra were calculated for a dielectric continuum mimicking pure water, *i.e.*, assuming *ε*_r_ ∼ 78 (presented in Fig. 9A), and also for corrected dielectric constants (Fig. 9B) to account for the screening ability of highly concentrated solutions. We further comment on the details of the spectra in the discussion section.

We performed the NBO analysis to obtain additional insight into the effects of the electrolyte on the electronic structure of the solvated water molecule. We performed the analysis for the 500 clusters extracted from the MD simulations for various concentrations. The clusters were embedded in the polarizable continuum with adjusted dielectric constants corresponding to neat water, 3 M and 8 M NaI solutions. The changes in electron densities of water were almost negligible for the 3 M solution (compared to neat water) while some effect of the electrolyte is observed for the 8 M NaI solution. The analysis showed that the occupation numbers of the oxygen lone pairs of the central water molecule are on average higher in the electrolyte – there is negligible charge transfer from the water lone pair to the sodium cation. The occupation numbers of the σ*(O–H) orbitals of the central water molecule are also little changed upon adding the electrolyte, with somewhat lower occupations in these orbitals (see the ESI, Fig. SI-6†). These observations are consistent with electrolyte-induced disruptions of the HB network. However, the observed approach of the 3a_1_ L and 3a_1_ H ionization energies on the one side and the 1b_2_ and 1b_1_ ionization energies can be traced to the stabilization of the lone pair electrons by sodium cations and destabilization of the electrons in the water bonding orbitals by iodide anions.

We also explored the effect of the environment and ions on the water ionization energies separately using idealized tetrahedral cluster models. These results may help to understand the molecular origin of the shifts in electron binding energies, and are discussed in more detail in the ESI.† The largest shifts in the BEs are found to be due to the interactions with sodium cations; the observed shifts are generally much larger than for the clusters obtained from the MD simulations. This is attributed to the local structure from the MD simulations rarely being ideally tetrahedral.

## 6. Discussion

In the following, we interpret our experimental liquid phase PE spectra with the help of the theoretical calculations. We first consider our experimental results. As described above (see ref. 67), without a fully characterized electric field between a sample liquid jet and an electron detector, global (sample-concentration-dependent) spectral shifts for given aqueous solutions may occur that cannot be accurately interpreted. However, it is possible to circumvent this effect by energetically aligning the spectra from the different NaI concentration samples at the positions of the spectrally isolated liquid water 1b_1_ peaks. Notably, such a treatment is only applicable when the entire PE spectrum experiences a uniform energetic shift, as if a bias voltage had been applied to the sample. As mentioned above, this is almost the case. Within this framework, peaks which do not align at different concentrations, as observed for the 1b_2_ and 3a_1_, identify orbitals that are affected by explicit water–water and ion–water interactions. Unfortunately, due to our spectral alignment methodology, the small spectral shifts that may also occur for the 1b_1_ energy cannot be captured in our analysis.

Initially we focus on the BEs of the solute ions, as extracted from Fig. 2–4 and SI-2–4† and summarized in Table 1. We find the BEs of the Na^+^ cation PE features to be independent of electrolyte concentration within our error bounds. In contrast, the anion BEs are observed to shift to slightly lower energies over the same concentration range. Comparing the vacuum/liquid interface and bulk-sensitive measurement results shown in Fig. 1 and 7, the concentration behavior of the solute BEs is found to be independent of our experimental probe depth. Significant differences between the relative intensities of the solute spectral features are observed between the vacuum/liquid-interface and bulk-sensitive data sets, however. These changes are due to the dependence of the partial ionization cross-sections and PE angular distributions on photon energy. Most prominent is the large increase of the I^–^ 4d to Na^+^ 2p intensity ratio, where the initially larger Na^+^ peak height becomes much smaller than the I^–^ peak height. This is primarily due to the steep increase of the I^–^ 4d anisotropy parameter, β, between 200 eV and 600 eV electron kinetic energy and our light polarization/electron detection geometry.^116^

Collectively analyzing the BE results extracted from different Na^+^I^–^ samples – including 0.5 M and 8.0 M aqueous solutions and crystalline solid NaI^113^ – we note near-identical intraspectral energetic separations between the Na^+^ 2p, I^–^ 4d, and I^–^ 5p PE features across environments. Additionally, despite the aqueous surface activity of I^–^,^124^–^127^ equivalent spectra are obtained from the aqueous vacuum/liquid-interface-sensitive and primarily bulk-sensitive data sets. This weak sensitivity to the solute surroundings is a clear indication of a minimal effect of solvation on the electronic structure of the ionic constituents.

In contrast to a previous investigation of the concentration dependence of aqueous alkali halide PE spectra,^127^ the I^–^ solute BEs reported here were found to vary slightly with electrolyte concentration (up to 150 ± 60 meV over a 7.5 M range). This discrepancy can likely be attributed to the lower energy resolution and the narrower concentration range adopted in the previous study. We note that under the previously adopted experimental conditions, the solute peak shifts reported here would be undetectable. The origin of the slight, I^–^ peak shifts observed here remain to be explored. However, one may speculate that these shifts are caused by the electrolyte-induced hydrogen-bonding network disruption and associated changes in charge donation by the polarizable I^–^ anions to the water antibonding, σ*(O–H), orbitals as the electrolyte concentration is increased.^18^ In contrast, the charge-dense Na^+^ ions are relatively unaffected as the electrolyte concentration increases, simply coordinating to the oxygen atoms of the solvent molecules in greater number. The theoretical NBO results support this interpretation, showing that the electron density of the sodium cation is unaffected as the electrolyte concentration is increased, while the iodide anion exhibits small charge transfer to water molecule.

In shifting our attention to ionic effects on the electronic structure of the liquid water solvent, we focus on the series of more liquid–vacuum-interface-sensitive spectra shown in Fig. 5. Here, spectral contamination from gas-phase water is minimized and trends in electrolyte concentration-dependent solvent spectral changes can be discerned. The observed shift to lower BE of the 1b_2_ feature relative to the 1b_1_ peak (of up to 370 ± 60 meV) with increasing electrolyte concentration is highlighted in Fig. 6. The apparent respective shifts to higher and lower BE of the 3a_1_ L and 3a_1_ H spectral components and increased spectral component overlap with increasing electrolyte concentration (differential component shift, Δ3a_1_, up to 450 ± 90 meV) is also highlighted in Fig. 6. In combination with the observation that the Na^+^ 2p, I^–^ 4d, and I^–^ 5p feature BEs are affected to a lesser degree by the increase in electrolyte concentration (see Fig. 2–4 and Table 2), these findings facilitate the following inferences. First, the 1b_2_ relative energetic shift with concentration can likely be considered an absolute energetic shift as the relative separations of the solute PE spectra features and the liquid water 1b_1_ feature all remain relatively unaltered over the studied concentration range. Given the opposite charges of the solute components, the coordination of the lone-pair 1b_1_ electrons to other water molecules or Na^+^ ions, and the intermolecular bonding character of the 1b_2_ orbital, the alternative explanation that the 1b_2_ PE feature remains fixed in energy while the solute and 1b_1_ solvent features shift is deemed unlikely. Second, the observation that the 1b_2_ and 3a_1_ PE features are discernibly affected as the electrolyte concentration is increased implies that any intermolecular water–water and water–ion interactions primarily involve these molecular orbitals.

The bulk-sensitive measurements shown in Fig. 7 reaffirm the aforementioned observations from the interfacially-sensitive data, albeit with an additional complication. Small differences in the behavior of the water 1b_2_ peak in the high photon energy, bulk-sensitive measurements may be partially obscured by additional spectral contributions from iodide (undetected in the 180/198 eV measurements) that overlap with this water peak at the high-BE side. This additional signal at 19.6 ± 0.4 eV BE (according to our fits) is attributed to ionization of the I^–^ 5s orbital, in agreement with the computed BE value of 20.2 ± 0.2 eV. Our assignment is also in accord with the expected ∼20% relative peak height compared to the I^–^ 4d signal intensity; here we have quantitatively accounted for the smaller ionization cross-section of I^–^ 5s compared to I^–^ 5p at a 650 eV photon energy, and we also considered the lower emission intensity for s-orbital ionization with respect to p-orbital ionization (based on values for atomic iodine^128^) in the adopted detection geometry, *i.e.*, with electron detection perpendicular to the light polarization. We have also accounted for the different electron occupancies. Despite the observed I^–^ 5s and water 1b_2_ spectral overlap, we were able to extract the 1b_2_ peak shift to lower BEs arising from the 0.5 M to 8.0 M NaI concentration increase. Based on multi-peak Gaussian fits to the I^–^ 5s and water 1b_2_ PE features, a 330 ± 60 meV 1b_2_ peak shift was extracted, in good agreement with the value (370 ± 60 meV) extracted over the larger concentration range (0.05 M to 8.00 M) associated with the interfacially-sensitive measurement results shown in Fig. 5. Using a similar analysis to that adopted for Fig. 5, we extracted a narrowing of the 3a_1_ L and 3a_1_ H peak splitting of 310 ± 120 meV from the lower concentration range data shown in Fig. 7. Considering our experimental uncertainties, this is consistent with the 450 ± 90 meV result extracted from the interfacially-sensitive spectra. On the other hand, the observed differences may arise from the different ion hydration structures at the surface and in the bulk. Unfortunately, our experimental uncertainties are too large to definitively identify any such surface and bulk hydration differences. Notably, differences are expected between surface and bulk hydration structures for such aqueous salt solutions,^129^ and these effects may contribute to the differences observed between the interfacially- and bulk-sensitive spectra.

As shown in Fig. 9, the calculated 1b_1_, 3a_1_, and 1b_2_ water valence peaks coincide well with the experimental results, although the theoretical peaks are narrower due to the classical nature of the MD simulations used in the present study.^31^ Small peaks in the 3a_1_ band at 12.2 eV or apparent shoulders in the spectra represent minor artefacts of the classical calculations. The theoretical spectra were shifted to lower energies by 0.3 eV in order to align those spectra representing neat water with the water 1b_1_ peak (11.16 eV BE). The simulations correctly reproduce the flat-top structure of the overlapping 3a_1_ H and 3a_1_ L peaks that are characteristic of the interatomic interactions predominantly described by 3a_1_ – 3a_1_ bonding (although the 1b_1_, 1b_2_ and 2a_1_ orbitals also contribute) between water molecules, as found experimentally for neat water. Furthermore, we qualitatively reproduce the change of the 3a_1_ peak shape as a function of concentration in the simulation. This change results from weakened 3a_1_ – 3a_1_ interactions upon addition of salt. Proton vibrations of the hydrogen-bond donor unit apparently modulate the electronic interaction, *i.e.*, the interaction is different for different minute geometric arrangements of the solution. When some of the associated water units are replaced by ions, the intermolecular bonding interaction is weakened, resulting in a narrowing of the 1b_1_ and 3a_1_ PE features, as observed experimentally. The electrolyte clearly disrupts the intermolecular electronic wave function overlap. The observed narrowing of the 3a_1_ BE peak is the most robust manifestation of ionic influence on the electronic and molecular structure of liquid water. It confirms that the unusual spectral shape of the 3a_1_ peak stems from hydrogen bonding; yet it does not result from the electrostatics but rather from the electronic interactions. Notably, similar ionic effects on the structure of liquid water are indicated by a range of other spectroscopies.^11^,^14^,^130^–^137^

Next, we discuss the energetic shifts of the calculated water BEs with increasing electrolyte concentrations in detail. We shall focus on the role of the dielectric continuum treatment in the calculations. The differences in the spectra are rather small but the effect is observable. In Fig. 9A, where the concentration dependence of the dielectric constant was neglected, we see that for the 3 M solution the 1b_1_ peak aligns with neat water and the 1b_2_ peak shifts by less than 0.1 eV to lower energies compared to neat water. For an 8 M solution, the 1b_1_ peak shifts to slightly higher energy (by 0.2 eV compared to neat water), and the 1b_2_ peak shifts to lower BEs by 0.2 eV compared to neat water. Interestingly, the relative energetic shift between the 1b_1_ and 1b_2_ peaks is close to that observed in the experiments. We can qualitatively understand this observation in terms of stabilization of the non-bonding 1b_1_ electron by the sodium cation, and destabilization of the 1b_2_ electron by the iodide anion when the dielectric constants are fixed to values associated with neat water. When accounting for concentration-dependent variations of *ε*_r_ and *ε*_opt_ (see the Method section), the 1b_1_ peak shift almost disappears; see Fig. 9B. Once we account for the variations of the effective dielectric constant with concentration, with *ε*_r_ (3 M) = 46 and *ε*_opt_ (3 M) = 1.86, and *ε*_r_ (8 M) = 22 and *ε*_opt_ (8 M) = 2.01, the 1b_1_ peaks line up closely, and only the 1b_2_ peak exhibits a small energetic shift of ∼0.34 eV to lower energies. Hence, these simulations are also in agreement with the experimental results. The spectra calculated with concentration-dependent dielectric constants correctly capture the BE peak positions, the structure of the peaks and their dependence on the electrolyte concentration. Contrary to the experiment, the theoretical calculations also provide the absolute energetics of the spectra. Spectra in Fig. 9B clearly show that the liquid water 1b_1_ peak position remains unaltered with increasing electrolyte concentration, which supports the experimental energetic referencing procedure. Indeed, collectively the experimental and theoretical results support the adoption of the spectrally isolated 1b_1(l)_ PE peak as a robust energetic reference for liquid-phase PE spectroscopy measurements.

The theoretical analysis presented in the ESI† shows that the main effect of the electrolyte is caused by the sodium cation. The iodide anion is much bigger than the sodium cation, its coordination distance is larger due to Pauli repulsion and consequently, the electrostatic interaction between the anion and the water is within the model smaller.

## 7. Conclusions

Most photoemission studies focus on how the solvent affects the solute. Here, we have explored the reverse, *i.e.*, the effects of electrolytes on water. We rely on the fact that the PE spectrum of water is very well known, and we can interpret even subtle variations. We observed that the electron binding energies of liquid water are only mildly affected even for a highly concentrated electrolyte solution. Our findings that the large change in the liquid structure, *i.e.*, the transformation from the dilute aqueous solution to the viscous almost crystalline-like phase, has so little effect on the water PE spectra are clearly surprising. Both the PE spectroscopy experiments and theoretical calculations show that the solute and solvent peak positions in the PE spectra are surprisingly stable with respect to increasing electrolyte concentrations. The directly observed variations are the small negative, relative energetic shifts of the I^–^ solute peaks (≤150 ± 60 meV) and water 1b_2_ (370 ± 60 meV) peak and the reduced spacing of the water 3a_1_ H and 3a_1_ L peak components (450 ± 90 meV). One important consequence from an experimental and practical viewpoint is that the present results fully justify the common procedure of aligning liquid-jet PE spectra from dilute or concentrated aqueous solutions using the well-resolved liquid water 1b_1_ peak. As the relative energetics of the solute peaks – that are energetically-referenced to the 1b_1_ peak – are found to be relatively insensitive to ion concentration, by extension the water 1b_1_ BE can also be inferred to be relatively insensitive to ion concentration. Through our analysis, we have determined an 150 ± 60 meV upper limit for the 1b_1_ BE shift over an ∼8 M solute concentration range. Given the large concentration range studied here, our results abate previous conjecture that an energetic shift of the water 1b_1_ PE peak position of up to 0.57 eV could be expected in going from (nearly) neat water to a 1 M NaCl aqueous solution.^138^ In defining still more robust energetic references for liquid PE spectroscopy experiments, the dielectric-constant-corrected PE spectra simulation results shown in Fig. 9B are encouraging. Indeed, the simulated few-tens-of-meV stability window of the water 1b_1_ BE is similar to the precisions with which liquid phase BEs can currently be determined. Further measurements dedicated to defining liquid water reference (1b_1_) and solute BEs, over large solute concentration ranges and with these high precisions, are ongoing in our laboratories.

It follows from the present work that the energetics of the individual ionizing transitions and the associated electrons do not change significantly during the solvation process – the relative BEs of the ions are essentially the same for dilute solutions, concentrated solutions, and crystalline NaI. This is consistent with the almost isoenthalpic nature of NaI dissolution. The solvation enthalpy for infinite dilution is only –7.53 kJ mol^–1^, and this number further decreases with increasing electrolyte concentration.^139^ As the total energy of the system varies little with electrolyte concentration, we cannot expect significant variations in the energetics of the electronic subsystems. On the other hand, the small variations in the BEs show that we are, in principle, able to investigate and detect the effects of solvation on the ionizing transitions and hence on the associated electrons.

Another crucial, although barely addressed question that arises regards the nature of PE spectroscopy's apparent insensitivity to the geometric structure of the aqueous solution. This behavior may surprise in the light of the X-ray PE spectroscopy technique's high sensitivity to a local atomic (chemical) environment which can lead to core-level (chemical) energy shifts of several electron volts.^54^,^140^ The insensitivity of the present spectra points toward the impressive screening ability of polar liquids and the more delocalized nature of the valence electrons on which we focus here. The experimental results presented indicate that aqueous-phase valence electron BEs are quite insensitive to purely electrostatic interactions between molecules over a wide range of concentrations. We can contrast this result with Auger electron energetics that have been shown to be much more sensitive towards ion pairing.^141^,^142^ On the other hand, aqueous-phase valence BEs have been shown to sensitively reflect changes in the covalent bonding of solute species. Valence binding energies of aqueous-phase PO_4_^3–^, HPO_4_^2–^ and H_2_PO_4_^–^ are, for example, very different while the BE of PO_4_^3–^ and its sodiated analogues remain almost identical.^42^ While adding NaCl to water will hardly change the binding energies of water molecules, a significant change will occur upon dissolving HCl in water; new covalently bonded species such as H_3_O^+^ emerge with completely different electronic structure characteristics. Comparatively, the electronic structure of water in electrolyte solutions is modified in a subtler way. Liquid water represents a collection of water molecules, which electronically interact within a shell of nearest neighbors. Upon increasing the electrolyte concentration, we disrupt these interactions and we tend towards “electronically isolated” water units embedded in a dielectric continuum.

Finally, an important finding of our work is the realization that the PE spectra of aqueous solutions can be reliably simulated using a relatively thrifty dielectric continuum approach. There are several important aspects. First, the nearest neighbors of the investigated water molecule have to be included in the calculations. Second, we need to focus only on the fully solvated water units. The IEDC approach is excellently suited for this purpose. The present technique does not require demanding *ab initio* MD calculations, and yet the results are reliable. Here, an important aspect is the proper treatment of the dielectric continuum. First, the non-equilibrium character of the ionization process has to be acknowledged.^37^,^89^ Second, we should adjust the dielectric continuum model to reflect the finite ionic strength of the studied solution. A number of studies that worked towards this goal have recently been published,^105^,^143^,^144^ and the present study is supportive of these concepts. Furthermore, these new aspects of photoemission data can be useful for testing classical MD simulations. Most of the employed force fields do not directly aim to describe highly concentrated solutions. The ability to describe the intermolecular bonding interactions that lead to the 3a_1_ water peak variations, which are the crucial experimentally observable quantities, can be utilized to develop improved dielectric continuum models. This would require investing some effort into getting the right results for the right reasons. For example, simulations taking into account the quantum character of the atomic nuclei and corresponding force fields should then be used.^31^,^145^ We also point out that the present data are relevant for the discussion of increased screening length at high electrolyte concentrations where Debye–Hückel theory is no longer applicable. A particularly interesting question is how the (Debye) decay length connects to the dielectric-scaled ion density; here the polarizability of the ions is expected to play a crucial role,^22^ signatures of which may be detectable in further liquid jet PE spectroscopy measurements and interpretable using the IEDC methodology.

## Conflicts of interest

## Supplementary Material

Supplementary information

## References

[cit1] Coffey P., Cathedrals of Science – The personalities and rivalries that made modern chemistry, Oxford University Press, 2008.

[cit2] Pettersson L. G. M., Henchman R. H., Nilsson A. (2016). Chem. Rev..

[cit3] Wernet P., Nordlund D., Bergmann U., Cavalleri M., Odelius M., Ogasawara H., Naslund L. A., Hirsch T. K., Ojamae L., Glatzel P., Pettersson L. G. M., Nilsson A. (2004). Science.

[cit4] Smith J. D., Cappa C. D., Wilson K. R., Messer B. M., Cohen R. C., Saykally R. J. (2004). Science.

[cit5] Huang C., Wikfeldt K. T., Tokushima T., Nordlund D., Harada Y., Bergmann U., Niebuhr M., Weiss T. M., Horikawa Y., Leetmaa M., Ljungberg M. P., Takahashi O., Lenz A., Ojamae L., Lyubartsev A. P., Shin S., Pettersson L. G. M., Nilsson A. (2009). Proc. Natl. Acad. Sci. U. S. A..

[cit6] Clark G. N. I., Cappa C. D., Smith J. D., Saykally R. J., Head-Gordon T. (2010). Mol. Phys..

[cit7] Henchman R. H., Cockram S. J. (2013). Faraday Discuss..

[cit8] Kühne T. D., Khaliullin R. Z. (2013). Nat. Commun..

[cit9] Harada Y., Miyawaki J., Niwa H., Yamazoe K., Pettersson L. G. M., Nilsson A. (2017). J. Phys. Chem. Lett..

[cit10] Gallo P., Arnann-Winkel K., Angell C. A., Anisimov M. A., Caupin F., Chakravarty C., Lascaris E., Loerting T., Panagiotopoulos A. Z., Russo J., Sellberg J. A., Stanley H. E., Tanaka H., Vega C., Xu L. M., Pettersson L. G. M. (2016). Chem. Rev..

[cit11] Bakker H. (2008). Chem. Rev..

[cit12] Jeyachandran Y. L., Meyer F., Nagarajan S., Benkert A., Bar M., Blum M., Yang W. L., Reinert F., Heske C., Weinhardt L., Zharnikov M. (2014). J. Phys. Chem. Lett..

[cit13] Yin Z., Inhester L., Veedu S. T., Quevedo W., Pietzsch A., Wernet P., Groenhof G., Föhlisch A., Grubmüller H., Techert S. (2017). J. Phys. Chem. Lett..

[cit14] Tielrooij K., Garcia-Araez N., Bonn M., Bakker H. (2010). Science.

[cit15] Omta A. W., Kropman M. F., Woutersen S., Bakker H. J. (2003). Science.

[cit16] Marcus Y. (2009). Chem. Rev..

[cit17] Yin Z., Rajkovic I., Kubicek K., Quevedo W., Pietzsch A., Wernet P., Föhlisch A., Techert S. (2014). J. Phys. Chem. B.

[cit18] Waluyo I., Nordlund D., Bergmann U., Schlesinger D., Pettersson L. G. M., Nilsson A. (2014). J. Chem. Phys..

[cit19] Suo L. M., Borodin O., Gao T., Olguin M., Ho J., Fan X. L., Luo C., Wang C. S., Xu K. (2015). Science.

[cit20] Yamada Y., Usui K., Sodeyama K., Ko S., Tateyama Y., Yamada A. (2016). Nat. Energy.

[cit21] Kuhnel R. S., Reber D., Battaglia C. (2017). ACS Energy Lett..

[cit22] Smith A. M., Lee A. A., Perkin S. (2016). J. Phys. Chem. Lett..

[cit23] Lee A. A., Perez-Martinez C. S., Smith A. M., Perkin S. (2017). Faraday Discuss..

[cit24] Goodwin Z. A. H., Kornyshev A. A. (2017). Electrochem. Commun..

[cit25] Sankari R., Ehara M., Nakatsuji H., Senba Y., Hosokawa K., Yoshida H., De Fanis A., Tamenori Y., Aksela S., Ueda K. (2003). Chem. Phys. Lett..

[cit26] Banna M. S., McQuaide B. H., Malutzki R., Schmidt V. (1986). J. Chem. Phys..

[cit27] Reutt J. E., Wang L. S., Lee Y. T., Shirley D. A. (1986). J. Chem. Phys..

[cit28] Page R. H., Larkin R. J., Shen Y. R., Lee Y. T. (1988). J. Chem. Phys..

[cit29] Truong S. Y., Yencha A. J., Juarez A. M., Cavanagh S. J., Bolognesi P., King G. C. (2009). Chem. Phys..

[cit30] Barth S., Ončák M., Ulrich V., Mucke M., Lischke T., Slavíček P., Hergenhahn U. (2009). J. Phys. Chem. A.

[cit31] Hollas D., Muchová E., Slavíček P. (2016). J. Chem. Theory Comput..

[cit32] Gaiduk A. P., Govoni M., Seidel R., Skone J. H., Winter B., Galli G. (2016). J. Am. Chem. Soc..

[cit33] Winter B., Weber R., Widdra W., Dittmar M., Faubel M., Hertel I. V. (2004). J. Phys. Chem. A.

[cit34] Nordlund D., Odelius M., Bluhm H., Ogasawara H., Pettersson L. G. M., Nilsson A. (2008). Chem. Phys. Lett..

[cit35] Nishizawa K., Kurahashi N., Sekiguchi K., Mizuno T., Ogi Y., Horio T., Oura M., Kosugi N., Suzuki T. (2011). Phys. Chem. Chem. Phys..

[cit36] Guo J. H., Luo Y. (2010). J. Electron Spectrosc. Relat. Phenom..

[cit37] Pluhařová E., Slavíček P., Jungwirth P. (2015). Acc. Chem. Res..

[cit38] Slavíček P., Winter B., Faubel M., Bradforth S. E., Jungwirth P. (2009). J. Am. Chem. Soc..

[cit39] Schroeder C. A., Pluhařová E., Seidel R., Schroeder W. P., Faubel M., Slavíček P., Winter B., Jungwirth P., Bradforth S. E. (2015). J. Am. Chem. Soc..

[cit40] Pluhařová E., Schroeder C., Seidel R., Bradforth S. E., Winter B., Faubel M., Slavíček P., Jungwirth P. (2013). J. Phys. Chem. Lett..

[cit41] Pluhařová E., Jungwirth P., Bradforth S. E., Slavíček P. (2011). J. Phys. Chem. B.

[cit42] Pluhařová E., Ončák M., Seidel R., Schroeder C., Schroeder W., Winter B., Bradforth S. E., Jungwirth P., Slavíček P. (2012). J. Phys. Chem. B.

[cit43] Debye P., Hückel E. (1923). Phys. Z..

[cit44] Fransson T., Harada Y., Kosugi N., Besley N. A., Winter B., Rehr J. J., Pettersson L. G. M., Nilsson A. (2016). Chem. Rev..

[cit45] Elles C. G., Rivera C. A., Zhang Y., Pieniazek P. A., Bradforth S. E. (2009). J. Chem. Phys..

[cit46] Elles C. G., Shkrob I. A., Crowell R. A., Bradforth S. E. (2007). J. Chem. Phys..

[cit47] Kerr G. D., Williams M. W., Birkhoff R. D., Hamm R. N., Painter L. R. (1972). Phys. Rev. A.

[cit48] Bernas A., Ferradini C., JayGerin J. P. (1997). Chem. Phys..

[cit49] Nilsson A., Nordlund D., Waluyo I., Huang N., Ogasawara H., Kaya S., Bergmann U., Naslund L. A., Ostrom H., Wernet P., Andersson K. J., Schiros T., Pettersson L. G. M. (2010). J. Electron Spectrosc. Relat. Phenom..

[cit50] Weinhardt L., Fuchs O., Blum M., Bär M., Weigand M., Denlinger J. D., Zubavichus Y., Zharnikov M., Grunze M., Heske C., Umbach E. (2010). J. Electron Spectrosc. Relat. Phenom..

[cit51] Winter B., Faubel M. (2006). Chem. Rev..

[cit52] Pietzsch A., Hennies F., Miedema P. S., Kennedy B., Schlappa J., Schmitt T., Strocov V. N., Föhlisch A. (2015). Phys. Rev. Lett..

[cit53] Nilsson A., Pettersson L. G. M. (2011). Chem. Phys..

[cit54] Winter B. (2009). Nucl. Instrum. Methods Phys. Res., Sect. A.

[cit55] Siegbahn H., Siegbahn K. (1973). J. Electron Spectrosc. Relat. Phenom..

[cit56] Lundholm M., Siegbahn H., Holberg S., Arbman M. (1986). J. Electron Spectrosc. Relat. Phenom..

[cit57] Delahay P., Von Burg K. (1981). Chem. Phys. Lett..

[cit58] Von Burg K., Delahay P. (1981). Chem. Phys. Lett..

[cit59] Delahay P., Dziedzic A. (1984). J. Chem. Phys..

[cit60] Seidel R., Winter B. and Bradforth S. E., in Annu. Rev. Phys. Chem., ed. M. A. Johnson and T. J. Martinez, 2016, vol. 67, pp. 283–305. 10.1146/annurev-physchem-040513-103715 27023757

[cit61] Faubel M., Steiner B., Toennies J. P. (1997). J. Chem. Phys..

[cit62] Salmeron M., Schlögl R. (2008). Surf. Sci. Rep..

[cit63] Starr D. E., Liu Z., Haevecker M., Knop-Gericke A., Bluhm H. (2013). Chem. Soc. Rev..

[cit64] Wu C. H., Weatherup R. S., Salmeron M. B. (2015). Phys. Chem. Chem. Phys..

[cit65] Winter B., Aziz E. F., Hergenhahn U., Faubel M., Hertel I. V. (2007). J. Chem. Phys..

[cit66] Kurahashi N., Karashima S., Tang Y., Horio T., Abulimiti B., Suzuki Y.-I., Ogi Y., Oura M., Suzuki T. (2014). J. Chem. Phys..

[cit67] There are two major sources of liquid jet charging, (1) electrokinetic charging, and (2) the low conductivity and charge screening capability of neat liquid water. As stated in the main body of the text, both of these effects can be mitigated by adding electrolyte to a solution to increase its conductivity, the latter effect can also be assuaged by decreasing the light intensity. In addition, a third effect (3), the possible change or evolution of the solution surface potential due to a molecular dipole layer must be taken into account. This effect is associated with the orientation of molecules at the water/aqueous solution – vacuum interface. A related effect is the possible larger surface propensity of one type of charged species (even an atomic ion such as iodide) compared to its counter ion, an effect that will give rise to an electrical double layer. It is perhaps useful to recall that molecular adsorption on a single-crystal surface (typically investigated by PE spectroscopy in ultra-high vacuum) is well known to cause changes of the sample’s work function. As of yet, for aqueous-solution PE spectroscopy, this effect has never been considered or detected. We note that any of the listed three effects will influence the kinetic energies of the photoelectrons (the position of the PE peak) that are measured in the experiment.

[cit68] Preissler N., Buchner F., Schultz T., Lübcke A. (2013). J. Phys. Chem. B.

[cit69] Kelly D. N., Lam R. K., Duffin A. M., Saykally R. J. (2013). J. Phys. Chem. C.

[cit70] It is perhaps interesting to mention that the accuracy of the reported water 1b_1_ BE may be challenged when realizing that inelastic scattering of the photoelectrons can lead to a measured (apparent) BE that differs from the actual (genuine) BE. Such effects have recently been observed in BE measurements of the hydrated electron in liquid jet photoelectron spectroscopy studies [Yamamoto *et al.*, *Phys. Rev. Lett.*, **112**, 2014, 187603 and Luckhaus *et al.*, *Sci. Adv.*, **3**, 2017, e1603224]. However, one must consider that these experiments adopted ionization photon energies of 3.6–13.6 eV, corresponding to relatively low electron kinetic energies of approximately 0–11 eV. Under such conditions, electron-phonon and electron-electron inelastic scattering cross-sections were found to be highly electron kinetic energy dependent. We note that in the experiments reported here, photoelectrons are produced and detected at significantly higher kinetic energies in the 120–193 eV and 590–645 eV ranges. At such kinetic energies, electron-electron inelastic scattering processes are expected to dominate, with inelastic scattering cross-sections and their energy dependences differing in the two probed electron kinetic energy ranges71 [Dingfelder *et al.*, *Radiat. Phys. Chem.*, **53**, 1998, 1]. Based on the calculated differential electron energy transfer cross-sections of liquid water at 100 eV and 500 eV, [Dingfelder *et al.*, *Radiat. Chem. Phys.*, **53**, 1998, 1] the apparent water 3a_1_ and 1b_2_ PE features may be expected to shift to lower BEs as the electron kinetic energy is increased from ∼160 eV to ∼620 eV. Such effects were not observed in the experimental data reported here, suggesting that such inelastic scattering effects have a, thus far, unmeasurable effect on aqueous valence electron BEs recorded using such high energy photons. In any event, the explicit treatment of such inelastic scattering and peak distortion effects requires more precise measurements of (electron kinetic energy dependent) inelastic mean free paths and inelastic scattering cross-sections in liquid water.^60^,^117^,^118^ Accordingly, such scattering effects and their influence on measured BEs are not considered explicitly in the present work, *i.e.*, we assume that the differential inelastic scattering cross-section is uniform over the electron kinetic energy and BE ranges considered here.

[cit71] Nguyen-Truong H. T. (2018). J. Phys.: Condens. Matter.

[cit72] Slavíček P., Winter B., Cederbaum L. S., Kryzhevoi N. V. (2014). J. Am. Chem. Soc..

[cit73] Hess B., Kutzner C., van der Spoel D., Lindahl E. (2008). J. Chem. Theory Comput..

[cit74] Berendsen H. J. C., Grigera J. R., Straatsma T. P. (1987). J. Phys. Chem..

[cit75] Parrinello M., Rahman A. (1981). J. Appl. Phys..

[cit76] Nose S. (1984). Mol. Phys..

[cit77] Hoover W. G. (1985). Phys. Rev. A.

[cit78] Hess B., Bekker H., Berendsen H. J. C., Fraaije J. (1997). J. Comput. Chem..

[cit79] Essmann U., Perera L., Berkowitz M. L., Darden T., Lee H., Pedersen L. G. (1995). J. Chem. Phys..

[cit80] Errington J. R., Debenedetti P. G. (2001). Nature.

[cit81] Chau P.-L., Hardwick A. (1998). Mol. Phys..

[cit82] Joung I. S., Cheatham T. E. (2008). J. Phys. Chem. B.

[cit83] Rubešová M., Muchová E., Slavíček P. (2017). J. Chem. Theory Comput..

[cit84] Bartlett R. J., Ranasinghe D. S. (2017). Chem. Phys. Lett..

[cit85] Levy M., Perdew J. P., Sahni V. (1984). Phys. Rev. A.

[cit86] Mulliken R. S. (1955). J. Chem. Phys..

[cit87] Della Sala F., Rousseau R., Gorling A., Marx D. (2004). Phys. Rev. Lett..

[cit88] Ončák M., Šištík L., Slavíček P. (2010). J. Chem. Phys..

[cit89] Rubešová M., Jurásková V., Slavíček P. (2017). J. Comput. Chem..

[cit90] Cossi M., Rega N., Scalmani G., Barone V. (2003). J. Comput. Chem..

[cit91] Barone V., Cossi M. (1998). J. Phys. Chem. A.

[cit92] Truong T. N., Stefanovich E. V. (1995). Chem. Phys. Lett..

[cit93] Jagoda-Cwiklik B., Slavíček P., Cwiklik L., Nolting D., Winter B., Jungwirth P. (2008). J. Phys. Chem. A.

[cit94] You Z. Q., Mewes J. M., Dreuw A., Herbert J. M. (2015). J. Chem. Phys..

[cit95] Guido C. A., Jacquemin D., Adamo C., Mennucci B. (2015). J. Chem. Theory Comput..

[cit96] Mewes J. M., You Z. Q., Wormit M., Kriesche T., Herbert J. M., Dreuw A. (2015). J. Phys. Chem. A.

[cit97] de Queiroz T. B., Kummel S. (2015). J. Chem. Phys..

[cit98] Boruah A., Borpuzari M. P., Kawashima Y., Hirao K., Kar R. (2017). J. Chem. Phys..

[cit99] Harris F. E., O'Konski C. T. (1957). J. Phys. Chem..

[cit100] Hasted J., Ritson D., Collie C. (1948). J. Chem. Phys..

[cit101] Haggis G., Hasted J., Buchanan T. (1952). J. Chem. Phys..

[cit102] Buchner R., Hefter G. T., May P. M. (1999). J. Phys. Chem. A.

[cit103] Winsor IV P., Cole R. H. (1982). J. Phys. Chem..

[cit104] Hasted J., Roderick G. (1958). J. Chem. Phys..

[cit105] Guan X. F., Ma M. M., Gan Z. C., Xu Z. L., Li B. (2016). Phys. Rev. E.

[cit106] Li B., Wen J. Y., Zhou S. G. (2016). Commun. Math. Sci..

[cit107] Clark H. A., Sutherland B. R. (2009). Exp. Fluids.

[cit108] Mennucci B., Cammi R., Tomasi J. (1998). J. Chem. Phys..

[cit109] Cossi M., Barone V. (2000). J. Phys. Chem. A.

[cit110] Glendening E. D., Landis C. R., Weinhold F. (2013). J. Comput. Chem..

[cit111] Reed A. (1988). Chem. Rev..

[cit112] Winter B., Weber R., Schmidt P. M., Hertel I. V., Faubel M., Vrbka L., Jungwirth P. (2004). J. Phys. Chem. B.

[cit113] Kowalczyk S. P., McFeely F. R., Ley L., Pollak R. A., Shirley D. A. (1974). Phys. Rev. B: Condens. Matter Mater. Phys..

[cit114] Lewis T., Winter B., Stern A. C., Baer M. D., Mundy C. J., Tobias D. J., Hemminger J. C. (2011). J. Phys. Chem. C.

[cit115] Lewis T., Faubel M., Winter B., Hemminger J. C. (2011). Angew. Chem., Int. Ed..

[cit116] Ottosson N., Faubel M., Bradforth S. E., Jungwirth P., Winter B. (2010). J. Electron Spectrosc. Relat. Phenom..

[cit117] Thürmer S., Seidel R., Faubel M., Eberhardt W., Hemminger J. C., Bradforth S. E., Winter B. (2013). Phys. Rev. Lett..

[cit118] Suzuki Y.-I., Nishizawa K., Kurahashi N., Suzuki T. (2014). Phys. Rev. E.

[cit119] Horinek D., Mamatkulov S. I., Netz R. R. (2009). J. Chem. Phys..

[cit120] Joung I. S., Cheatham T. E. (2009). J. Phys. Chem. B.

[cit121] Jensen K. P., Jorgensen W. L. (2006). J. Chem. Theory Comput..

[cit122] Di Tommaso D., Ruiz-Agudo E., de Leeuw N. H., Putnis A., Putnis C. V. (2014). Phys. Chem. Chem. Phys..

[cit123] Hartkamp R., Coasne B. (2014). J. Chem. Phys..

[cit124] Jungwirth P., Tobias D. J. (2001). J. Phys. Chem. B.

[cit125] Jungwirth P., Tobias D. J. (2002). J. Phys. Chem. B.

[cit126] Liu D. F., Ma G., Levering L. M., Allen H. C. (2004). J. Phys. Chem. B.

[cit127] Ottosson N., Heyda J., Wernersson E., Pokapanich W., Svensson S., Winter B., Öhrwall G., Jungwirth P., Björneholm O. (2010). Phys. Chem. Chem. Phys..

[cit128] Yeh J.-J., Atomic Calculations of Photoionization Cross Sections and Asymmetry Parameters, Gordon and Breach, Langhorne, PA, 1993.

[cit129] Jungwirth P., Tobias D. J. (2006). Chem. Rev..

[cit130] Smith J. D., Saykally R. J., Geissler P. L. (2007). J. Am. Chem. Soc..

[cit131] Wang Y., Tominaga Y. (1994). J. Chem. Phys..

[cit132] Mizoguchi K., Ujike T., Tominaga Y. (1998). J. Chem. Phys..

[cit133] Amo Y., Tominaga Y. (1998). Phys. Rev. E.

[cit134] Ujike T., Tominaga Y., Mizoguchi K. (1999). J. Chem. Phys..

[cit135] Foggi P., Bellini M., Kien D. P., Vercuque I., Righini R. (1997). J. Phys. Chem. A.

[cit136] Tielrooij K., Van Der Post S., Hunger J., Bonn M., Bakker H. (2011). J. Phys. Chem. B.

[cit137] O'Brien J. T., Williams E. R. (2012). J. Am. Chem. Soc..

[cit138] Olivieri G., Goel A., Kleibert A., Cvetko D., Brown M. A. (2016). Phys. Chem. Chem. Phys..

[cit139] Madelung O., New series group III, 1987, vol. 22, 63, p. 117.

[cit140] Hüfner S., Photoelectron Spectroscopy: Principles and Applications, Springer-Verlag, Berlin, Heidelberg, New York, London, Paris, Tokyo, Hong Kong, Barcelona, Budapest, 1995.

[cit141] Pohl M. N., Richter C., Lugovoy E., Seidel R., Slavíček P., Aziz E. F., Abel B., Winter B., Hergenhahn U. (2017). J. Phys. Chem. B.

[cit142] Unger I., Seidel R., Thürmer S., Pohl M. N., Aziz E. F., Cederbaum L. S., Muchová E., Slavíček P., Winter B., Kryzhevoi N. V. (2017). Nat. Chem..

[cit143] Lange A. W., Herbert J. M. (2011). J. Chem. Phys..

[cit144] Frecer V., Miertus S. (1992). Int. J. Quantum Chem..

[cit145] McBride C., Vega C., Noya E. G., Ramirez R., Sese L. M. (2009). J. Chem. Phys..

