# Supplementary material for: Do water's electrons care about electrolytes?
Source: Chem Sci. 2018 Nov 1;10(3):848–65. doi: 10.1039/c8sc03381a (PMC6346409; doi:10.1039/c8sc03381a)
Supplement: Supplementary file 1 [file SC-010-C8SC03381A-s001.pdf]

Electronic Supplementary Material (ESI) for Chemical Science.
This journal is © The Royal Society of Chemistry 2018

Supporting Information

Do water's electrons care of electrolytes?

Marvin N. Pohl,[1,2] Eva Muchová,[3] Robert Seidel,[4,5] Hebatallah Ali,[1,2] Štěpán Sršeň,[3] Iain Wilkinson,[4] Bernd Winter,[1,*] and Petr Slavíček [3,*]

[1] *Fritz-Haber-Institut der Max-Planck-Gesellschaft, Faradayweg 4-6, D-14195 Berlin, Germany Berlin, Germany*

[2] *Fachbereich Physik, Freie Universität Berlin, Arnimallee 14, D-14195 Berlin, Germany*

[3] *Department of Physical Chemistry, University of Chemistry and Technology, Technická 5, 16628 Prague, Czech Republic*

[4] *Helmholtz-Zentrum Berlin für Materialien und Energie, Hahn-Meitner-Platz 1, D-14109 Berlin, Germany*

[5] *Humboldt-Universität zu Berlin, Department of Chemistry, Brook-Taylor-Str. 2, D-12489 Berlin, Germany*

[*] *Corresponding author: iain.wilkinson@helmholtz-berlin.de, winter@fhi-berlin.mpg.de and petr.slavicek@vscht.cz*

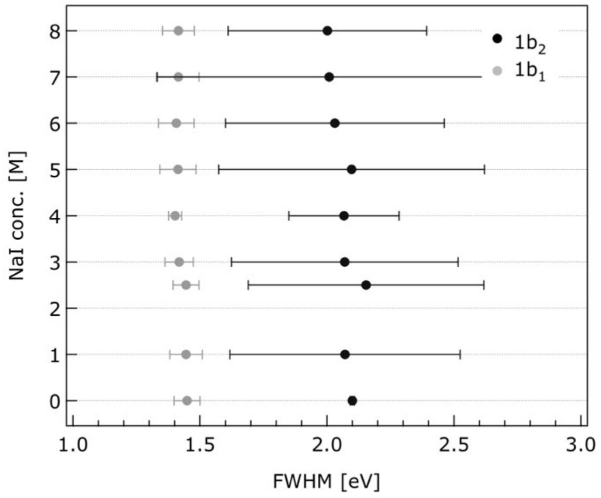

Figure SI-1 Evolution of the $1b_1$ and $1b_2$ peak full-width at half-maximum (FWHM) in NaI aqueous solutions as a function of concentration, 0.05 M to 8.0 M. All data were extracted from Figure 5. The error bars represent the uncertainties of the fitting procedure.

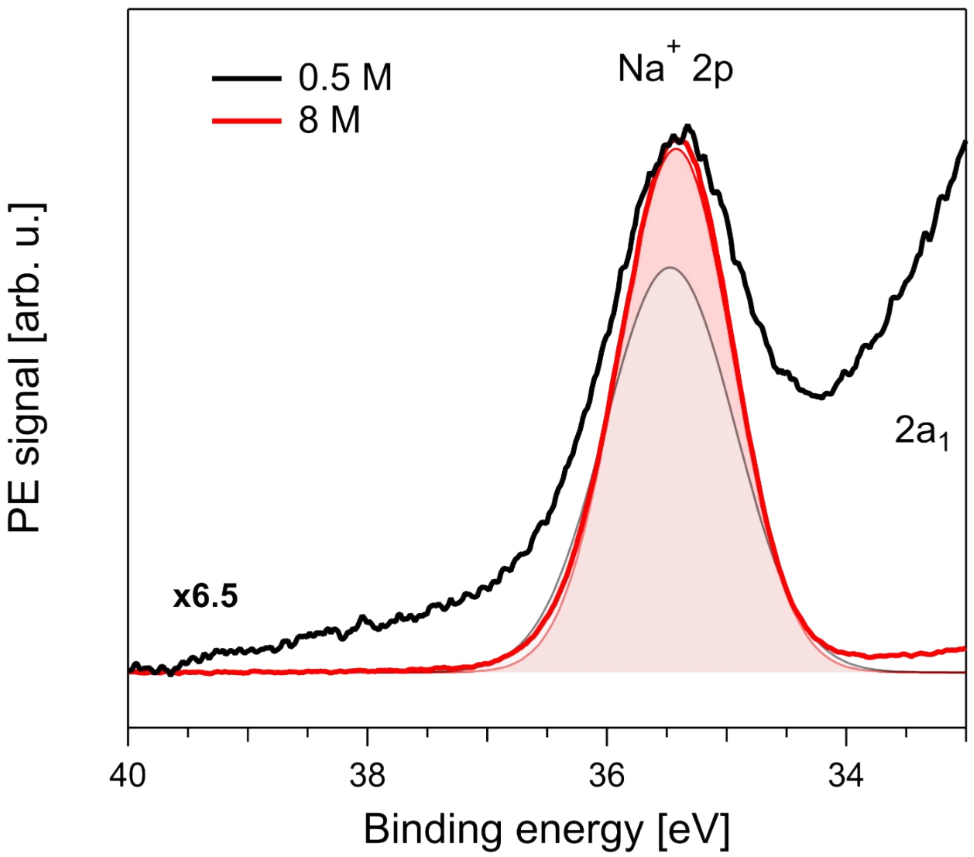

Figure SI-2 Enlarged view of the Na^+ 2p and water $2a_1$ photoelectron spectra of 0.5 M (black) and 8.0 M (red) NaI (aq) solutions measured at a 650 eV photon energy. The intensity of the 0.5 M concentration spectrum was multiplied by a factor of 6.5 to yield the same peak height as that observed from the 8.0 M solution. Gaussian fits of the Na^+ 2p peak are presented (the fits in black and red correspond to the 0.5 M and 8.0 M solutions, respectively). Spectral contributions primarily associated with water $2a_1$ ionization have been subtracted.

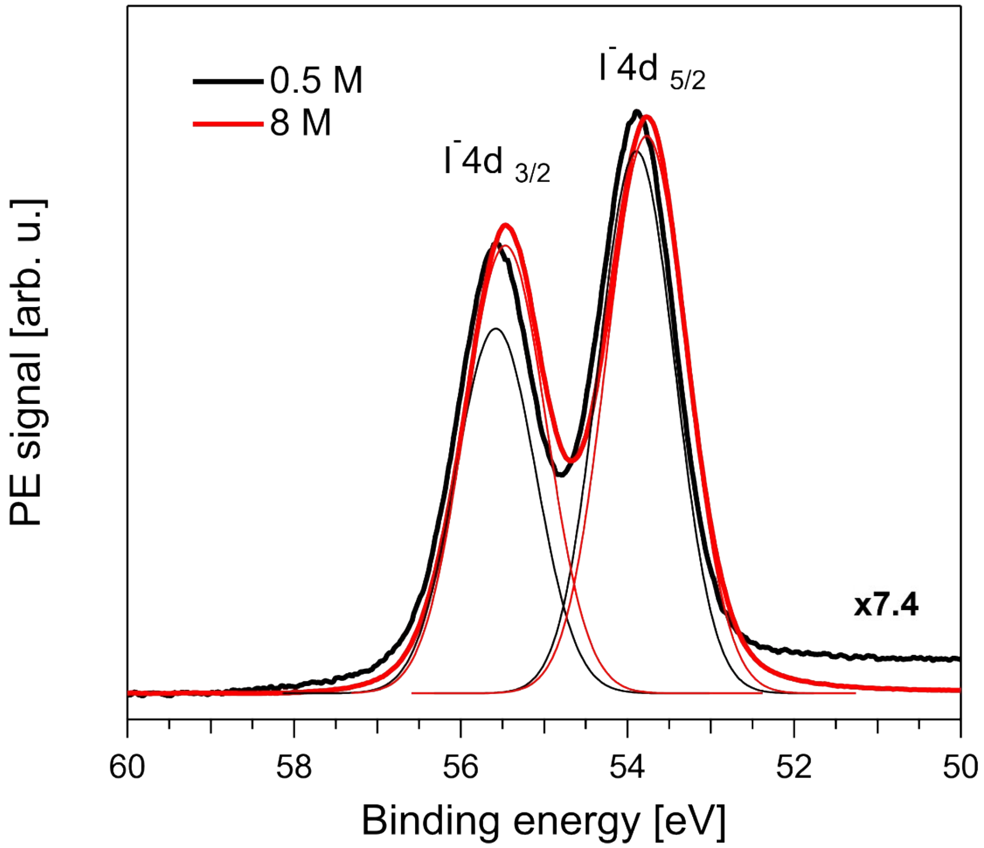

Figure SI-3 Enlarged view of the I^- 4d $_{5/2}$ and I^- 4d $_{3/2}$ photoelectron spectra of 0.05 M (black) and 8.0 M (red) NaI (aq) solutions measured at a 650 eV photon energy. The intensity of the 0.5 M concentration spectrum was multiplied by a factor of 7.4 to yield the same peak heights as those observed from the 8.0 M solution. Gaussian fits of the I^- 4d $_{5/2}$ and water I^- 4d $_{3/2}$ components are presented (the fits in black and red correspond to the 0.5 M and 8.0 M solutions, respectively).

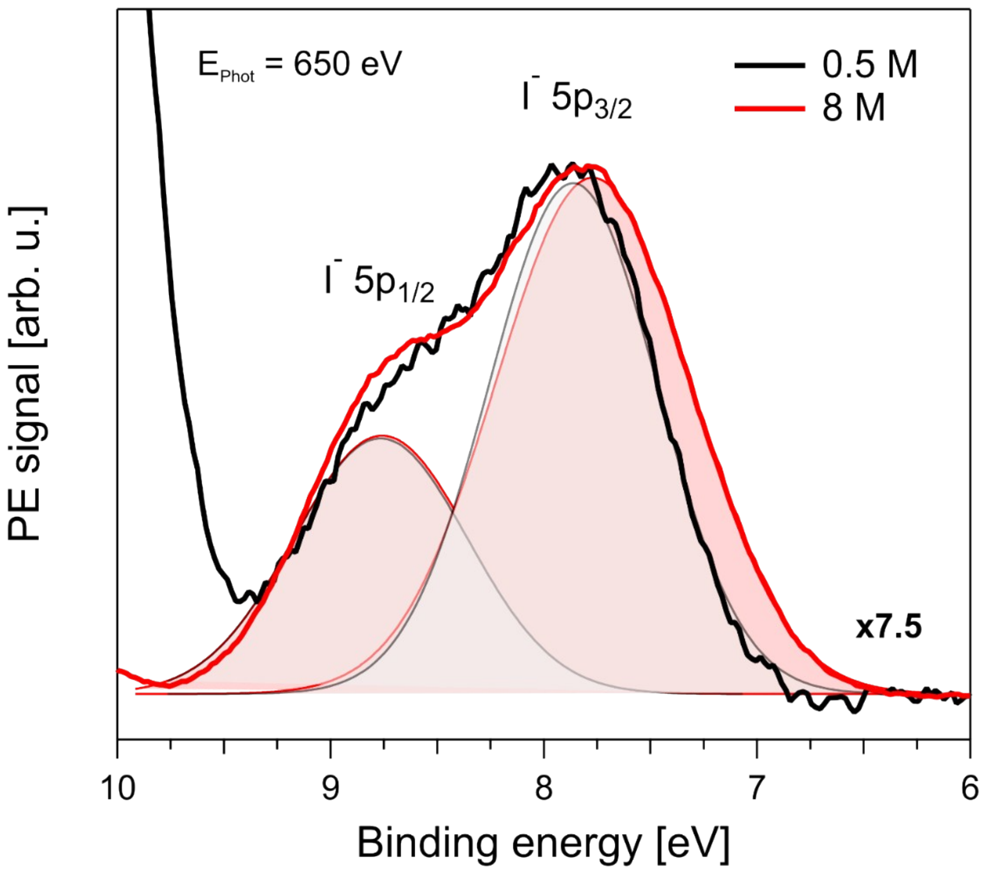

Figure SI-4 Enlarged view of the I^- 5p $_{3/2}$ and I^- 5p $_{1/2}$ photoelectron spectra of 0.05 M (black) and 8.0M (red) NaI (aq) solutions measured at a 650 eV photon energy. The intensity of the 0.5 M concentration spectrum was multiplied by a factor of 7.5 to yield the same peak heights as those observed from the 8.0 M solution. Gaussian fits of the I^- 5p $_{3/2}$ and water I^- 5p $_{1/2}$ components are presented (the fits in black and red correspond to the 0.5 M and 8.0 M solutions, respectively).

Ionic effects in small model clusters

We separately explored the effect of the environment and ions on the ionization energies for idealized cluster models. Figure SI-5 and Table SI-1 show the ionization energies of water units for the following situations (i) pure water pentamer cluster, (ii) one iodide anion replaces one of the HB-accepting water units (iodide anion was positioned at the distance O–I^- of 3.6 Å), (iii) one sodium cation replaces one of the HB-donating water units (sodium cation was positioned at the distance O–Na^+ of 2.3 Å), or (iv) both a sodium cation and an iodide anion replace a pair of water units (ions were positioned at distances O–Na^+ of 2.3 Å and O–I^- of 3.6 Å). The selected bond lengths for O–I^- and O–Na^+ correspond to the first peak in the respective radial distribution functions. The clusters were not optimized, because it would inevitably lead to distorted structures irrelevant for the discussion. In Table SI-1, the rows show the BEs for the cluster. The fraction by which the central water molecule contributes to each orbital from which we ionize is given in parentheses. We can infer from the table and figure that the effect of ions is most pronounced in the gas-phase clusters, especially if the charge is not compensated by a counterion. The shift can be close to 5 eV if water is replaced by a sodium cation. Yet the values change only slightly (tenths of an eV) when both sodium and iodide are coordinated to a central water molecule. Embedding the pentamers in a dielectric continuum causes a strong shielding effect, which leads to much smaller energy changes upon the replacement of water by the ions. The main effect on the water binding energies can be attributed to the sodium cation (approximately 1eV); the iodide anion leaves the BEs essentially unchanged (the change is 0.1 eV). When both ions are present, the water BEs are again significantly shifted to higher energies. Such large shifts in BEs were not observed for clusters extracted from MD, but it was shown that the majority of local structural motives do not correspond to the idealized tetrahedral structure. Upon increasing the ion – water distances, e.g. the ion is more loosely bound to the cluster, the energy shift decreases.

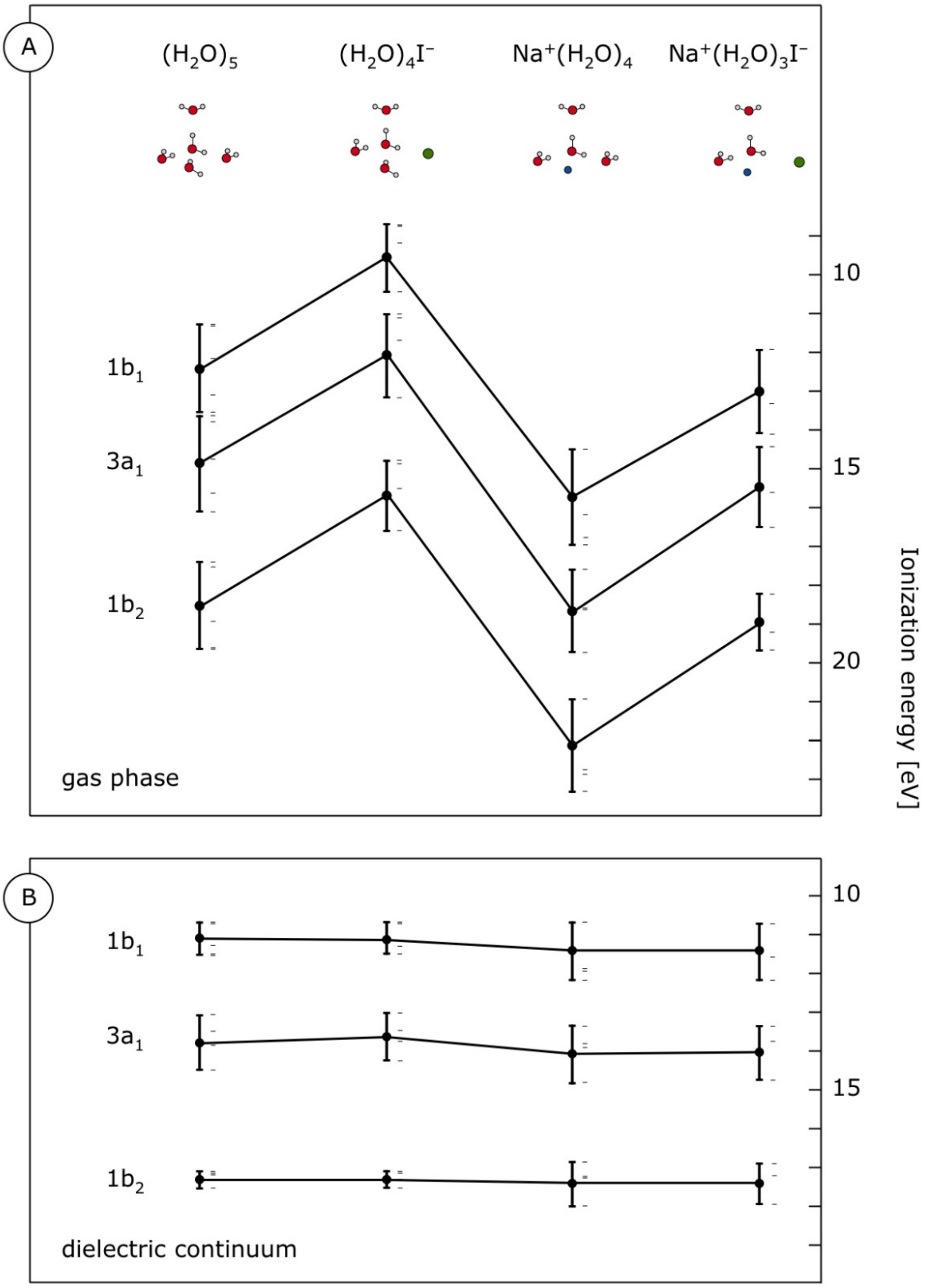

Figure SI-5 Binding energies of the electrons associated with the central water unit in a model water cluster. The central water molecule is surrounded by four water units. The results for the water pentamer are shown in the left-most data points in the Figure. Moving from left to right, a hydrogen bond donor unit is then replaced by a sodium cation, a hydrogen bond acceptor unit is replaced by an iodide anion, and a hydrogen bond donor and a hydrogen bond acceptor are replaced by a sodium cation and an iodide anion, respectively. Panel A shows the data for a cluster in the gas phase, panel B shows the data for a similar cluster embedded in a dielectric continuum. The plotted data were taken from Table SI-1.

$(H_2O)_5$		$(H_2O)_4 \ldots I^-$			$(H_2O)_4 \ldots Na^+$			$(H_2O)_3 \ldots Na^+ \ldots I^-$	
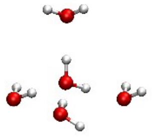		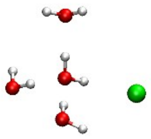			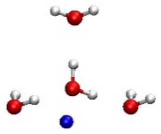			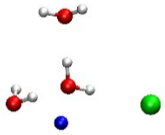	
gas phase	diel.cont.	gas phase	diel.cont.	diel.cont.	gas phase	diel.cont.	diel.cont.	gas phase	diel.cont
		R(O-I$^-$) 3.6 Å	R(O-I$^-$) 3.6 Å	R(O-I$^-$) 4.5 Å	R(O-Na$^+$) 2.3 Å	R(O-Na$^+$) 2.3 Å	R(O-Na$^+$) 3.5 Å	R(O-Na$^+$), R(O-I$^-$) 2.3 Å, 3.6 Å	
11.27(0.00)	10.71(0.07)	8.70(0.00)	10.71(0.04)	10.69(0.02)	14.48(0.01)	10.72(0.00)	10.64(0.00)	11.90(0.00)	10.71(0.00)
11.32(0.01)	10.63(0.00)	8.72(0.02)	10.68(0.00)	10.70(0.00)	16.20(0.20)	11.93(0.20)	11.04(0.86)	13.31(0.52)	11.57(0.14)
12.25(0.65)	11.24(0.74)	9.22(0.77)	11.31(0.74)	11.44(0.71)	16.73(0.04)	11.88(0.05)	11.41(0.13)	14.10(0.04)	12.18(0.67)
13.09(0.19)	11.48(0.11)	10.45(0.29)	11.46(0.11)	11.53(0.14)	16.97(0.04)	12.20(0.63)	11.49(0.09)	14.42(0.46)	13.34(0.26)
13.61(0.09)	11.54(0.07)	10.96(0.06)	12.99(0.24)	13.02(0.22)	17.62(0.75)	13.35(0.26)	13.09(0.39)	15.62(0.54)	13.71(0.19)
13.62(0.05)	13.05(0.23)	11.10(0.17)	13.49(0.14)	13.52(0.15)	18.68(0.29)	13.89(0.23)	13.52(0.22)	16.47(0.35)	14.72(0.69)
13.82(0.28)	13.50(0.12)	11.70(0.54)	13.68(0.24)	13.69(0.23)	18.86(0.08)	13.83(0.05)	13.75(0.06)	18.17(0.11)	16.88(0.08)
14.76(0.50)	13.76(0.23)	13.16(0.12)	14.24(0.42)	14.30(0.45)	19.76(0.43)	14.86(0.58)	14.42(0.30)	19.14(0.20)	17.20(0.01)
15.63(0.08)	13.75(0.06)	14.80(0.04)	17.05(0.04)	17.08(0.04)	20.94(0.23)	16.85(0.07)	16.68(0.05)	19.72(0.72)	17.95(0.93)
16.10(0.11)	14.51(0.35)	14.89(0.08)	17.13(0.08)	17.16(0.08)	22.69(0.00)	17.28(0.00)	17.14(0.73)		
17.44(0.05)	17.07(0.04)	15.51(0.85)	17.30(0.24)	17.19(0.07)	22.90(0.02)	17.31(0.01)	17.31(0.00)		
17.58(0.11)	17.15(0.08)	16.56(0.04)	17.51(0.77)	17.50(0.88)	23.29(0.90)	17.99(0.92)	17.38(0.19)		
18.94(0.88)	17.35(0.24)								
19.69(0.01)	17.14(0.00)								
19.71(0.00)	17.55(0.67)								

Table SI-1 Calculated ionization energies of water for the idealized tetrahedral water-ion model structures (data for BEs of iodide and sodium ions are not provided). The model clusters comprise four situations - (i) pure water pentamer cluster, (ii) one iodide anion replaces one of the hydrogen bond-accepting water units, (iii) one sodium cation replaces one of the hydrogen bond-donating water units, or (iv) both a sodium cation and an iodide anion replace a pair of water units. The rows of the table show the BEs for the cluster. The fraction by which the central water molecule contributes to each orbital from which we ionize is given in parentheses. As we mostly focus on the central water molecule, the most relevant BEs are the ones for which the fraction is the highest, these are also plotted in Figure SI-5. For $(H_2O)_4 \ldots I^-$ and $(H_2O)_4 \ldots Na^+$ clusters we also considered two distances between the ion and the central water oxygen atom; one corresponding to the first peak of the respective RDF curve, the second corresponding to the ion weakly bound to the cluster.

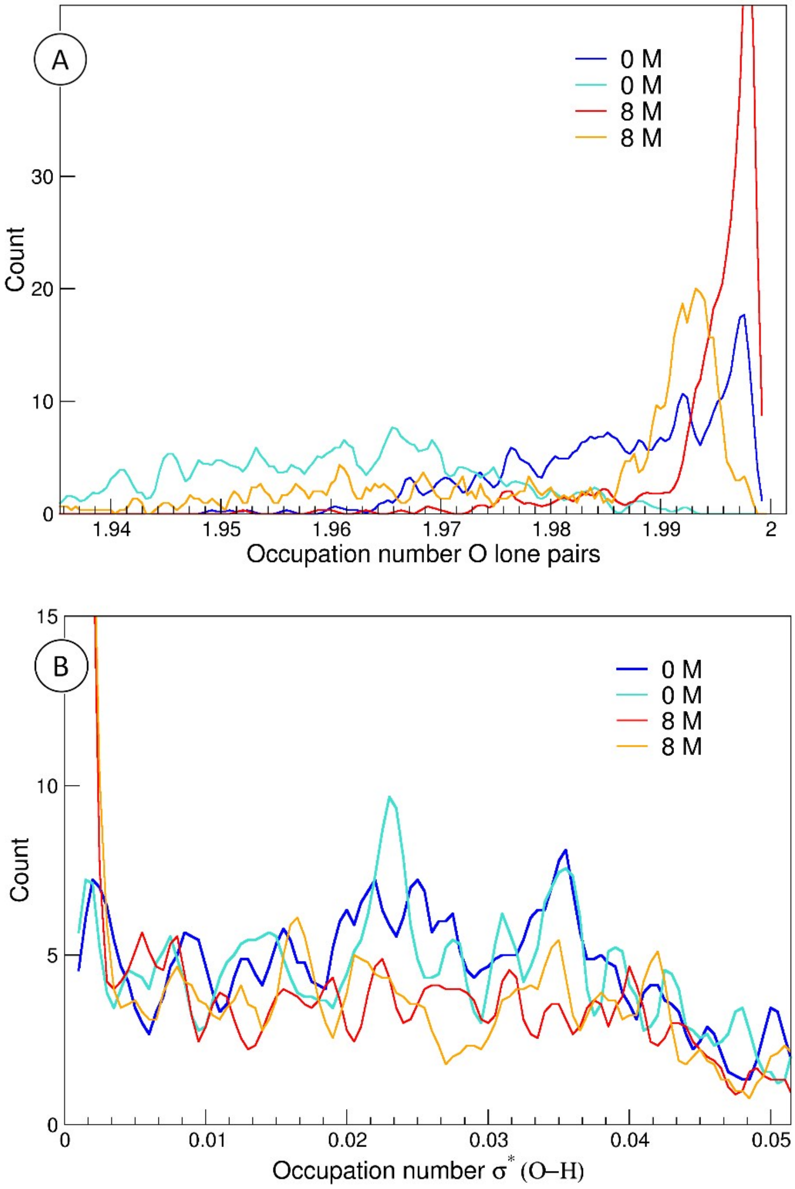

Figure SI-6 Histogram showing the occupation numbers of the two oxygen lone pairs (A) and two σ^*(O-H) orbitals (B) of the central water molecule for 500 structures extracted from the MD simulations for neat water (0 M) and for 8 M NaI solution. The figure shows that the occupation numbers of the oxygen lone pairs of the central water molecule are higher in the electrolyte, e.g. there is negligible charge transfer from the water lone pair to the sodium cation. The occupation numbers of the two σ^*(O-H) orbitals of the central water molecule are somewhat lower compared to neat water. For a fraction of the central water molecules, the occupation number in the two σ^*(O-H) orbitals is almost zero.
